# Limited evidence for quantitative contribution of rare and endangered species to agricultural production

**DOI:** 10.1016/j.agee.2022.108326

**Published:** 2023-04-01

**Authors:** Vivienne P. Groner, Jessica J. Williams, Richard G. Pearson

**Affiliations:** Department of Genetics, Evolution and Environment, Centre for Biodiversity and Environment Research, University College London, Gower Street, London WC1E 6BT, United Kingdom

**Keywords:** Agriculture, Biodiversity, Conservation, Ecosystem services

## Abstract

Biodiversity underpins ecosystem functions that provide benefits to people, yet the role of rare and endangered species (RES) in supporting ecosystem services is unclear. Thus, it remains controversial whether arguments for conservation that focus on ecosystem services align with the protection of RES. We designed a systematic review protocol to critically assess the evidence for quantitative contributions of RES to terrestrial agricultural production, which is a key driver of biodiversity change and, simultaneously, could suffer from the loss of ecosystem services provided by biodiversity. Our review search criteria required that studies: 1) provide information on RES, 2) focus on an ecosystem service relevant for agriculture; and 3) include a quantitative measure of agricultural production. Surprisingly, we found only four studies that fulfilled these criteria, which was insufficient to perform a meta-analysis of results. Thus, we highlight here the gap in quantitative research, discuss the implications of this knowledge gap for the conservation of RES, and suggest future research directions. We conclude that further quantitative research is urgently needed to better inform conservation and agricultural policies, including research that focuses specifically on RES, incorporates more ecosystem services, and covers a wider range of climatic and socioeconomic contexts.

## Highlights

• Literature search for rare & endangered species’ (RES) role in agricultural production.
• Current evidence is too scarce to perform a meta-analysis.
• It remains unclear how RES conservation aligns with provision of ecosystem services.
• Further quantitative research is needed to better inform agricultural policies.

## 1 Introduction

In recent decades justification for biodiversity conservation has shifted from species’ intrinsic value towards a focus on the preservation of ecosystem services and the benefits they provide to people (Reid et al., 2005, Mace, 2014, IPBES, 2019, Dasgupta and McKenzie, 2020). This trend has raised concerns because it remains unclear to what extent rare and endangered species (RES) play important roles in providing ecosystem services, and therefore whether arguments focused on benefits to people are sufficient to justify conservation of RES (McCauley, 2006, Kleijn et al., 2015, Pearson, 2016). Species can be defined as rare or endangered due to small population sizes, low population densities, small geographical ranges, restricted habitat types, or a combination of all these (Rabinowitz, 1981, Lyons et al., 2005, IUCN, 2021). RES have been shown to contribute to key provisioning, supporting, and cultural ecosystem services that are of direct benefit to people (Dee et al., 2019, Mouillot et al., 2013); for example, services provided by RES include wildlife watching of rare birds (Booth et al., 2011), medicinal or ornamental plants for personal use or as income-earning opportunities (Groner et al., 2022), and goods that gain value with increasing rarity such as sturgeon caviar (Gault et al., 2008). Moreover, several studies have inferred that RES play a role in ecosystem processes that underpin services such as carbon cycling (Fauset et al., 2015) and crop pollination (Kremen et al., 2002, MacLeod et al., 2020, Winfree et al., 2018). However, there is a need to synthesise knowledge of how RES provide quantifiable contributions to ecosystem service provision. If RES are shown to contribute significantly to ecosystem services, conservation arguments focused on benefits to people would align with the protection of RES. However, if a small number of common species provide most of ecosystem services, as suggested by some studies (e.g., Grime, 1998; Smith and Knapp, 2003; Winfree et al., 2015; Lohbeck et al., 2016), then conservation actions that focus on maintaining ecosystem services will offer little benefit to RES protection (Dee et al., 2019).

Agriculture is a key driver of biodiversity change (Sala et al., 2000, Reid et al., 2005) and, at the same time, could suffer from the loss of species (IPBES, 2019, IUCN, 2021). Thus, arguments for conservation based on ecosystem services have become a focus of agricultural policies (e.g. the UK Agricultural Act 2020; Coe and Finlay, 2020). Food demands continue to increase with the growing world population (van Dijk et al., 2021). As we strive for sustainable land use through changes in food consumption and diet (FAO, 2018; ‘2030 Agenda for Sustainable Development’, United Nations, 2015), it is crucial that there is a strong evidence-base to support arguments for conservation that are centred on ecosystem services. Aiming to synthesise knowledge that fills this gap, we designed a systematic review protocol to 1) assess the strength of evidence of the quantitative contribution of RES to agricultural production, and 2) identify areas where further research may be needed. However, due to a scarcity of research that fulfilled our review criteria, we could not perform a meta-analysis of results. Here, we highlight the gap in quantitative research, the implications of this gap in knowledge for the conservation of RES, and future research directions.

## 2 Methods

This section summarises the key criteria of the systematic search we designed to address our research question. The full systematic review protocol and a detailed description of the criteria are available in Appendix S1.

### 2.1 Search strategy

We performed a comprehensive search of the scientific literature adhering to the ‘Guidelines for Systematic Review in Conservation and Environmental Management’ (Pullin and Stewart, 2006). In July 2021 (and updated in November 2022), we searched two electronic databases: SCOPUS and Web of Science Core Collection (WOS). Each search string was composed of three variables: 1) a synonym of ‘rare’ or ‘endangered’, or the name of an endangered species; 2) an ecosystem service relevant for agricultural production; and 3) a quantitative measure of agricultural production. We limited the search to peer-reviewed studies published up until November 2022 in English, German, French, Dutch, or Spanish language.

### 2.2 Inclusion criteria

A full description of the pre-specified inclusion and exclusion criteria is provided in Appendix S1 (Section S1.2 and Table S1). We kept our inclusion criteria purposefully strict to focus on quantitative research. In summary, we focused on terrestrial animals, plants, microbes, and fungi that are described in the literature as ‘rare’, ‘endangered’, ‘vulnerable’, ‘threatened’, or with ‘restricted or declining area’, or ‘restricted or declining population’ following IUCN Red List criteria (IUCN, 2021). In addition to the criteria-based search, we looked for endangered species published in the IUCN Red List of European bees (Nieto et al., 2014), the IUCN Red list of Bird and Mammal pollinators (Regan et al., 2015), and the Xerces Society Red List of Pollinating Insects of North America (National Research Council, 2007). We included studies on ecosystem services that can be performed by the included species and are categorised as relevant for food and agriculture by the FAO (DuVal et al., 2019), consistent with the Millennium Ecosystem Assessment report (Reid et al., 2005): climate regulation, natural-hazard regulation, pest and disease regulation, pollination, nutrient cycling, soil formation, water cycling, and habitat provisioning. We did not include primary production as an ecosystem service in the search because we were focusing on those RES that are providing services for agriculture, rather than being the agricultural product themselves. As measures of RES contribution, we accepted studies that presented quantitative (e.g., crop biomass per ha) or monetary (e.g., dollars per ha) assessments of agricultural production.

### 2.3 Screening, quality check, and data extraction

We followed an independent double-screening approach (Appendix S1: Section S1.3) to eliminate documents that did not meet the pre-specified inclusion criteria (see Section 2.2 and Appendix S1: Section S1.2). To be considered as being of satisfactory quality, the paper had to report 1) a control experiment, 2) at least one replicate, and 3) uncertainties associated with quantitative results (e.g., Yanai et al., 2021). From the studies that met inclusion and quality criteria, we extracted the following information: type of study and region, species, type of rarity (e.g., low abundance, IUCN status), ecosystem services, and contribution to agricultural production.

## 3 Results

**Fig. 1:**
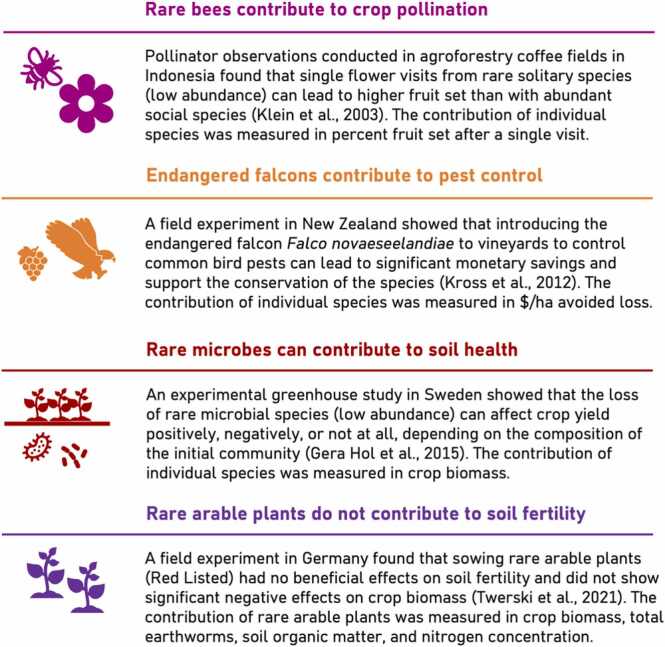
Studies identified in the systematic review that provided quantitative measures of the contribution of rare or endangered species (RES) to agricultural production.

The systematic search identified 2943 unique citations of which only four studies met all the inclusion criteria (Fig. 1). Across these four studies, the focal species (and study location) were bees (Indonesia), microbes (Sweden), arable plants (Germany), and birds (New Zealand). Two studies considered endangered species according to the Red List; the other studies defined rare as ‘low in abundance’. The ecosystem services investigated were pollination (1), pest control (1), and soil fertility (2); one study also reported the absence of a dis-services. One study provided a monetary estimate of the quantitative contribution ($ per ha), two studies measured crop biomass, and one study estimated the probability of fruit set after a single pollinator visit.

From the included studies, we found mixed evidence for RES contribution to agricultural production and the data showed no patterns related to the type of ecosystem service. Two studies suggested that the contribution of RES is of quantitative importance (Klein et al., 2003, Kross et al., 2012), one study reported no effect of RES and highlighted the absence of a dis-service (Twerski et al., 2021), and one study was inconclusive (Gera Hol et al., 2015).

Further, we found twelve studies that did not fulfil all the criteria for our systematic review but are relevant to the debate as to whether arguments focused on benefits to people align with the protection of RES (Chen et al., 2020, Hędrzak et al., 2021, Kleijn et al., 2015, Kremen et al., 2002, MacLeod et al., 2020, Simpson et al., 2022, Soliveres et al., 2016, Staton et al., 2022, Sutter et al., 2017, Winfree et al., 2015, Winfree et al., 2018, Zhang et al., 2022). For example, MacLeod et al. (2020) studied the overlap in identity and flower preferences between regionally rare species and dominant pollinators in United States (following Kleijn et al.'s (2015) definition of a dominant crop pollinator as a species that accounts for at least 5% of the total number of individual bees collected from a given crop) and found that 19% of dominant crop pollinators were regionally rare, which supports the idea that RES can be important providers of ecosystem service. The study was excluded because it did not provide a quantitative contribution of RES to agricultural production. Soliveres et al. (2016) studied the relative functional importance of rare and common species in driving the biodiversity-multifunctionality relationship in grasslands. They suggest that locally rare above-ground species are the most important diversity component to preserve high levels of ecosystem multifunctionality in managed grasslands, perhaps due to their lower proportion of negative functional effects. In line with this study, Chen et al. (2020) and Zhang et al. (2022) show that rare below-ground species drive ecosystem multifunctionality. All three studies were excluded because they did not quantify an agricultural product. A detailed description of the additional studies and justification for exclusions are provided in Appendix S1: Section S2.

## 4 Discussion

To our knowledge, this was the first systematic review that aimed to assess the quantitative contribution of RES to agricultural production. Considering the conversation around the conservation of species for the ecosystem services they provide and/or their intrinsic value, in both the scientific community (Dee et al., 2017, Kleijn et al., 2015) and in recent politics (e.g. UK Agricultural Act 2020; Coe and Finlay, 2020), it is surprising that we found only four studies that fulfilled the criteria despite an extensive search strategy. Based on the small number of available studies, we conclude that arguments based on ecosystem services currently lack a strong evidence base to support the conservation of RES.

Despite conducting a thorough review of the literature, there may have been studies looking at the quantitative contribution of RES that we missed. For example, because we were interested in the role of RES in supporting agricultural production, we did not include studies on RES and their contribution to agriculture or the food system more broadly through primary productivity. Although our systematic search of the published literature covered two extensive databases and multiple languages, we might have missed studies published in other databases, grey literature, or in other languages. Further, we considered only a subset of ecosystem services (see Methods; Reid et al., 2005) and excluded marine systems. Other processes that contribute indirectly to agricultural production were not included in our systematic review, for example biotic interactions (Cardinale et al., 2002, Wright et al., 2017) and regulation of local climate through biophysical and biogeochemical processes (Foley et al., 2003, Groner et al., 2018).

The small number of available studies highlights the need for more research to understand the role of RES in ecosystem services in the context of agricultural production. We identify three areas for future research. First, studies of ecosystem services in agricultural landscapes should focus specifically on RES. Studies of RES are particularly difficult because such species are less likely to be observed performing a service than are dominant or abundant species. RES contributions may also not have been picked up in previous studies because they can be highly context dependent and vary across space (Loreau et al., 2003) and time (Yachi and Loreau, 1999). This could be partly addressed with longer and more frequent observations. Further, RES may be more difficult to identify because researchers might be less familiar with their specific traits and classify them only to family level. Such specific traits could be crucial to understand RES contribution to ecosystem function and resilience (e.g., Diaz et al., 2013; Jain et al., 2014). This could be overcome with expert training or the use of multiple methods, for example human observation in combination with video recordings (e.g., Frank et al., 2007). Second, studies of the role of RES in agricultural landscapes should include a broader range of ecosystem services and taxonomic groups. We found that the literature is heavily biased towards pollination services and insects as service providers. Exploring the interactions of ecosystem services could add another level of complexity to the system (Bennett et al., 2009, Garibaldi et al., 2018); for example, it has been shown that pest control can boost crop yield due to increased insect pollination (Lundin et al., 2012, Sutter and Albrecht, 2016). Large-scale mapping of species-based ecosystem services could benefit from a better understanding of RES' contribution and a more appropriate representation of RES in weighted provider richness (Ceaușu et al., 2021). Third, future studies should consider a broader range of climatic and socioeconomic contexts. We found that studies tended to be biased towards wealthy countries with good data availability. In the future, studies should aim to reach a larger spatial coverage to explore the role of RES in agricultural systems of different climatic and socioeconomic contexts. This includes the effects of farm size (e.g., smallholder vs commercial), farming practices (e.g., organic vs conventional), and level of intensification.

## CRediT authorship contribution statement

**VG and RP** conceptualised the study; **VG** performed online search, analysis, and led paper writing with input from all authors; **VG and JW** screened articles.

## Declaration of Competing Interest

The authors declare that they have no known competing financial interests or personal relationships that could have appeared to influence the work reported in this paper.

## Data Availability

All data that support the findings of this study are referenced in the article.
