# Supplementary material for: Limited evidence for quantitative contribution of rare and endangered species to agricultural production
Source: Agric Ecosyst Environ. 2023 Apr 1;345:108326. doi: 10.1016/j.agee.2022.108326 (PMC10485819; doi:10.1016/j.agee.2022.108326)
Supplement: Supplementary file 1 — Supplementary material [file mmc1.docx]

**Appendix S1: Limited evidence of a quantitative contribution of rare and endangered species to agricultural production**

Vivienne P. Groner, Jessica J. Williams, and Richard G. Pearson

# **S1 Systematic review protocol**

**S1.1 Search strategy**

We performed a comprehensive search of the scientific literature adhering to the ‘Guidelines for Systematic Review in Conservation and Environmental Management’ (Pullin & Stewart, 2006). On 7 July 2021 (and updated 4 November 2022), we searched two electronic data bases: SCOPUS and Web of Science Core Collection (WOS). Each search string was composed of three variables: 1) a synonym of ‘rare’ or ‘endangered’, or the name of an endangered species; 2) an ecosystem service relevant for agricultural production; and 3) a quantitative measure of agricultural production. We limited the search to peer-reviewed studies published up until November 2022 (primary research papers and reviews included, no pre-registered studies, no grey literature, no updated search) in English, German, French, Dutch, or Spanish language.

**S1.2 Inclusion and exclusion criteria**

An overview over the pre-specified inclusion criteria is provided in Table S1. We kept our inclusion criteria purposefully strict to focus on quantitative research. We focused on terrestrial animals, plants, microbes, and fungi that are described in the literature as ‘rare’, ‘endangered’, ‘vulnerable’, ‘threatened’, or with ‘restricted or declining area’, or ‘restricted or declining population’ following IUCN Red List criteria (IUCN, 2021). In addition to the criteria-based search, we looked for endangered species published in the IUCN Red List of European bees (Nieto et al., 2014), the IUCN Red list of Bird and Mammal pollinators (Regan et al., 2015), and the Xerces Society Red List of Pollinating Insects of North America (National Research Council, 2007). We excluded aquatic organisms, terrestrial wild animals and plants that are consumed as wild meat or wild vegetables, wetland species, peatland species, livestock and cattle, and other managed species.

We included studies on ecosystem services that can be performed by the included species and are categorised as relevant for food and agriculture by the FAO (DuVal et al., 2019), consistent with the Millennium Ecosystem Assessment report (Reid et al., 2005): climate regulation, natural-hazard regulation, pest and disease regulation, pollination, nutrient cycling, soil formation, water cycling, and habitat provisioning. We excluded studies on non-food primary production, waste decomposition and detoxification, non-food raw materials (e.g., biofuel and fibres), genetic resource, carbon sequestration, disease regulation in livestock, medicine, and cultural ecosystem services because these services are not directly linked to quantifiable agricultural outputs. We did also not include primary production as an ecosystem service in the search because we were focusing on those RES that are providing services for agriculture, rather than being the agricultural product themselves.

As measures of RES contribution, we accepted studies that presented quantitative (e.g., crop biomass) or monetary (e.g., dollars per ha) assessments of agricultural production. We excluded studies of heavily managed production whereby there is little scope for contribution of wild species (e.g., livestock, honey production), as well as studies in which the agricultural output could not be associated with a single RES (or group of RES). We did not attempt to infer contribution of non-dominant species from publications that presented only the contribution of the most common or dominant species. For pollination studies, we did not accept flower visitation rate as a quantitative contribution to agricultural production because it has been shown to be a poor predictor of pollination (King et al., 2013)*.*

Table S1: Inclusion and exclusion criteria for the selection of relevant literature on rare or endangered species’ contribution to agricultural production.

|  | **Included** | **Excluded** |
| --- | --- | --- |
| **Years** | Up to July 2021 | After July 2021 |
| **Languages** | English, German, French, Dutch, Spanish | other |
| **Rare/endangered** | terrestrial animals,  terrestrial plants,  terrestrial microbes and fungi | aquatic organisms,  terrestrial wild animals and plants that are consumed as wild meat or wild vegetables,  wetland and peatland species,  livestock and cattle,  managed species |
| **Ecosystem service** | climate regulation,  natural hazard regulation,  pest and disease regulation, pollination,  nutrient cycling,  soil formation,  water cycling,  habitat provisioning | primary production,  waste decomposition and detoxification,  raw material,  genetic resource,  carbon sequestration,  disease regulation in livestock, medicine,  cultural ecosystem services |
| **agricultural production** | Quantitative  Cereal crops,  fruit and vegetables,  coffee and cacao  and others | Qualitative  Cereal crops, fruit and vegetables,  coffee and cacao,  or  Livestock and cattle,  Flowers, Honey, Energy crops,  Aquatic food sources |

**S1.3 Screening and data extraction**

One author (VG) screened titles and abstracts of all search results to eliminate documents that did not meet the pre-specified inclusion criteria. For studies identified as potentially relevant, two authors (VG and JW) considered the full text independently, and any disagreement was resolved in discussion. One author (VG) extended the search to the reference lists of included articles as well as publication records of the authors of included articles until July 2021 (Table S7).

Once the list of included studies was completed, two authors (VG and JW) independently extracted the following information from the included studies: type of study and region, species, type of rarity (e.g., low abundance, IUCN status), ecosystem services, and contribution to agricultural production. Inconsistencies were discussed as necessary.

We identified four main reasons for the exclusion of articles, which often occurred in combination: 1) studies were clearly off-topic (e.g. studies on bee allergies); 2) studies presented the effects of environmental change on biodiversity or RES but not on ecosystem services or functions; 3) studies presented effects of species richness, species groups, or of the most common species only (e.g. honeybee - *Apis mellifera*) when quantifying species contribution to ecosystem services; and 4) the results were not quantitative.

**S1.4 Study quality**

All included studies were assessed for quality based on three pre-defined criteria. To be considered as being of satisfactory quality, the paper had to report 1) a control experiment, 2) at least one replicate, and 3) uncertainties associated with quantitative results (e.g. Yanai et al., 2021).

**S1.5 Data analysis**

Due to the heterogeneity of included studies, we could not perform a statistical meta-analysis. Instead, we followed guidance from the Cochrane handbook (Deeks et al., 2019), which supports the use of a narrative approach when meta-analysis is inappropriate. We grouped the results according to their conclusion on the relevance of RES for agricultural production.

# **S2 Results**


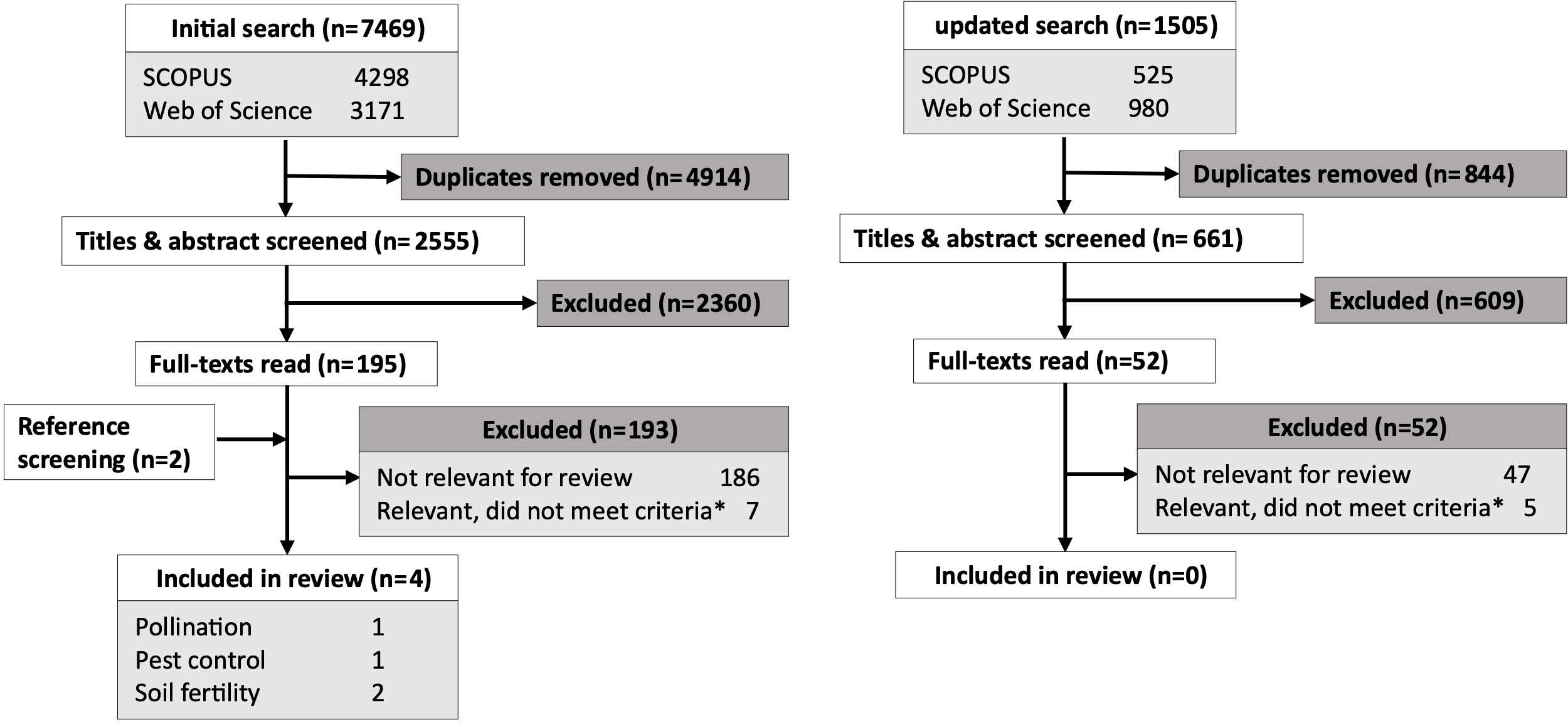


*Figure S1: Flow diagram illustrating the number of studies in each step for the initial search in July 2021 and the updated search in November 2022. In total, we identified 2943 unique citations. * We found twelve studies that did not fulfil all our criteria (no agricultural context and/or no quantitative results) but are highly relevant to debate as to whether arguments focused on benefits to people align with the protection of RES, see section below.*

**Additional important studies that did not fulfil all inclusion criteria**

We found twelve studies that did not fulfil all the criteria for our systematic review but are highly relevant to the debate as to whether arguments focused on ecosystem services align with the protection of RES. The studies did not fulfil the criteria because they did not quantify the contribution of RES to agricultural production.

Of those studies, eight suggested that the contribution of RES is of importance. A pollinator observation experiment conducted in watermelon fields in the United States (Kremen et al., 2002) and a comparison of watermelon, cranberry, and blueberry fields in the United States (Winfree et al., 2018) agreed in their conclusion that rare (low abundance) wild bees substantially contribute to pollination service. Further, they discussed the importance of rare species for crop stability with respect to species turnover and suggested that the presence of rare species is essential to reach the pollination threshold for optimal crop yield. Both studies were excluded because they did not provide quantitative contributions of RES. MacLeod et al. (2020) studied the overlap in identity and flower preferences between regionally rare species and dominant pollinators in United States (following Kleijn et al.'s (2015) definition of a dominant crop pollinator as a species that accounts for at least 5% of the total number of individual bees collected from a given crop) and found that 19% of dominant crop pollinators were regionally rare, which supports the idea that RES can be important providers of ecosystem service. The study was excluded because it did not provide a quantitative contribution of RES to agricultural production. Soliveres et al. (2016) studied the relative functional importance of rare and common species in driving the biodiversity-multifunctionality relationship in grasslands. They suggest that locally rare above-ground species are the most important diversity component to preserve high levels of ecosystem multifunctionality in managed grasslands, perhaps due to their lower proportion of negative functional effects. In line with this study, Chen et al., (2020) and Zhang et al., (2022) show that rare below-ground species drive ecosystem multifunctionality. All three studies were excluded because they did not quantify an agricultural product. Simpson et al., (2022) used data from 11 plant-bee visitation networks in New Jersey, USA, to show that on average across plant communities, 25% of bee species that were important at the community scale were also numerically rare within their network. The study was excluded because it was not set in an agricultural production context. Hędrzak et al., (2021) reviewed contributions of the common hamster, an endangered species, to services in agricultural ecosystems and provide an overview of methods to quantify the contribution. The study was excluded because it did not go beyond the conceptual quantification.

In contrast, three studies concluded that the contribution of RES is relatively unimportant. A study that synthesised 90 pollination data sets from around the globe and estimated the $ per crop contribution of individual species showed that a few common species provided most of the service while the majority of species made a contribution of less than 5% (Kleijn et al., 2015). Regionally threatened species were rarely observed on crops (no corresponding contribution was presented). From this the authors concluded that pollination service seems to be an insufficient argument for conservation. Winfree et al. (2015) came to a similar conclusion using an analytical approach (Price equation; Fox, 2006) to partition the contribution to pollination of watermelon, cranberry, and blueberry fields made by species richness, composition and abundance in four large-scale data sets in the United States. The study showed that abundance fluctuations of dominant species drive ecosystem service delivery, whereas richness changes are relatively unimportant because they primarily involved rare species that contributed little to function. The study was excluded because it did not provide a quantitative contribution by RES. An assessment of pollinator species richness, species diversity, and functional trait diversity between agroforestry fields and paired monoculture arable controls in eastern England showed that nationally rarer bee species also contributed substantially to functional richness but not consistently to functional dispersion. This suggests that while they provide a unique functional role, their contributions to ecosystem services remain limited by low local abundances (Staton et al., 2022). This study was excluded because agricultural production was not quantified.

The remaining study was unclear on the role of RES. Based on an analysis of plant-pollinator networks in herbaceous systems in Switzerland, Sutter et al. (2017) showed that rare bees tended to visit nested subsets of plant species that were also visited by crop pollinators. This is an important contribution to the understanding of the overlapping resource requirements among RES and service providing species, which is crucial for effective measures that unify management for multiple objectives. However, because the authors did not quantify the pollination effort of studied bees, we could not categorize RES contribution in our study.

# **S3 Search terms**

## Search terms and strings - English

Table S2: Search terms for systematic literature search on rare or endangered species’ contribution to agricultural production in English. The asterisk (*) was used to find variations of words with a common root. For the advanced search strings, we connected entries of each column with ‘OR’ and combined columns with ‘AND’.

| **Rare/endangered** | | **Ecosystem service** | **Contribution to**  **agricultural production** |
| --- | --- | --- | --- |
| *IUCN*  rare  endangered  vulnerable  threatened | | ecosystem service*  ecosystem function*  *regulating services*  climate regulat*  natural-hazard regulat*  pest regulat*  disease regulat*  pollin*  *supporting services*  nutrient cycl*  soil form*  water cycl*  habitat provis* | farm*  agri*  food*  *General contribution*  contribut*  produc*  return*  profit*  revenue  return  output  incom*  *Unit measure contribution*  kg  ton*  $ / dollar  kcal / calories  ha / hectare  bushel  *Food contribution*  crop  harvest*  yield |
| low  small  few  limit*  restrict*  confin*  declin*  decreas*  fall*  loss | *area*  range  distribution  extent  niche  habitat  *abundance*  number  population*  individuals  density |  |  |
| *Species name-based search:*  Red list European bees (N=77)  Red list pollinating birds (N=169)  Red list pollinating mammals (N=101)  Red list pollinating insects of North America (N=111) | |  |  |

## SCOPUS - English

### IUCN

#### General contribution

**- 293 documents**

TITLE-ABS-KEY (IUCN OR rare OR "endanger*" OR vulnerable OR "threaten*")

AND TITLE-ABS-KEY (“ecosystem servic*” OR “ecosystem function*”)

AND TITLE-ABS-KEY (“farm*” OR “agri*” OR “food”)

AND TITLE-ABS-KEY ("contribut*" OR "produc*" OR "return*" OR "profit*" OR revenue OR return OR output OR "incom*")

AND (EXCLUDE (PUBYEAR, 2022)) AND (LIMIT-TO (LANGUAGE, “English”))

AND NOT (aquatic OR marine OR livestock OR cattle OR pig OR bushmeat OR “wetland*” OR “peatland*” OR “medicin*”)

**- 279 documents**

TITLE-ABS-KEY (IUCN OR rare OR "endanger*" OR vulnerable OR "threaten*")

AND TITLE-ABS-KEY (“pest regulat*” OR “disease regulat*” OR “pollin*” OR “nutrient cycl*” OR “soil form*” OR “climate regulat*” OR “natural-hazard regulat*” OR “water cycl*” OR “habitat provis*”)

AND TITLE-ABS-KEY (“farm*” OR “agri*” OR “food”)

AND TITLE-ABS-KEY ("contribut*" OR "produc*" OR "return*" OR "profit*" OR revenue OR return OR output OR "incom*")

AND (EXCLUDE (PUBYEAR, 2022)) AND (LIMIT-TO (LANGUAGE, “English”))

AND NOT (aquatic OR marine OR livestock OR cattle OR pig OR bushmeat OR “wetland*” OR “peatland*” OR “medicin*”)

#### Unit measure contribution

**- 75 documents**

TITLE-ABS-KEY (IUCN OR rare OR "endanger*" OR vulnerable OR "threaten*")

AND TITLE-ABS-KEY (“ecosystem servic*” OR “ecosystem function*” OR “pest regulat*” OR “disease regulat*” OR “pollin*” OR “nutrient cycl*” OR “soil form*” OR “climate regulat*” OR “natural-hazard regulat*” OR “water cycl*” OR “habitat provis*”)

AND TITLE-ABS-KEY (“farm*” OR “agri*” OR “food”)

AND TITLE-ABS-KEY (kg OR tonnes OR "$" OR "dollar*" OR kcal OR calories OR hectare OR "ha" OR “ton*” OR “bushel*”)

AND (EXCLUDE (PUBYEAR, 2022)) AND (LIMIT-TO (LANGUAGE, “English”))

AND NOT (aquatic OR marine OR livestock OR cattle OR pig OR bushmeat OR “wetland*” OR “peatland*” OR “medicin*”)

#### Food contribution

**- 365 documents**

TITLE-ABS-KEY (IUCN OR rare OR "endanger*" OR vulnerable OR "threaten*")

AND TITLE-ABS-KEY (“ecosystem servic*” OR “ecosystem function*” OR “pest regulat*” OR “disease regulat*” OR “pollin*” OR “nutrient cycl*” OR “soil form*” OR “climate regulat*” OR “natural-hazard regulat*” OR “water cycl*” OR “habitat provis*”)

AND TITLE-ABS-KEY (“farm*” OR “agri*” OR “food”)

AND TITLE-ABS-KEY (crop OR "harvest*" OR yield)

AND (EXCLUDE (PUBYEAR, 2022)) AND (LIMIT-TO (LANGUAGE, “English”))

AND NOT (aquatic OR marine OR livestock OR cattle OR pig OR bushmeat OR “wetland*” OR “peatland*” OR “medicin*”)

### AREA

#### General contribution

**- 307 documents**

**TITLE-ABS-KEY ("small distribution" OR "small range" OR "small extent" OR "small niche" OR "small habitat" OR "small area" OR "limit*distribution" OR "limit* range" OR "limit* extent" OR "limit* niche" OR "limit* habitat" OR "limit* area*" OR "restrict*distribution" OR "restrict* range" OR "restrict* extent" OR "restrict* niche" OR "restrict* habitat" OR "restrict* area*" OR "confin* distribution" OR "confin* range" OR "confin* extent" OR "confin* niche" OR "confin* habitat" OR "confin* area*" OR "declin* distribution" OR "declin* range" OR "declin* extent" OR "declin* habitat" OR "declin* niche" OR "declin* habitat" OR "declin* area" OR "decreas* distribution" OR "decreas* range" OR "decreas* extent" OR "decreas* habitat" OR "decreas* niche" OR "decreas* habitat" OR "decreas* area" OR ("loss " AND "distribution") OR ("loss " AND "range ") OR ("loss " AND "extent") OR ("loss " AND "niche") OR ("loss" AND "habitat"))**

AND TITLE-ABS-KEY (“ecosystem servic*” OR “ecosystem function*”)

AND TITLE-ABS-KEY (“farm*” OR “agri*” OR “food”)

AND TITLE-ABS-KEY ("contribut*" OR "produc*" OR "return*" OR "profit*" OR revenue OR return OR output OR "incom*")

AND (EXCLUDE (PUBYEAR, 2022)) AND (LIMIT-TO (LANGUAGE, “English”))

AND NOT (aquatic OR marine OR livestock OR cattle OR pig OR bushmeat OR “wetland*” OR “peatland*” OR “medicin*”)

**- 251 documents**

**TITLE-ABS-KEY ("small distribution" OR "small range" OR "small extent" OR "small niche" OR "small habitat" OR "small area" OR "limit*distribution" OR "limit* range" OR "limit* extent" OR "limit* niche" OR "limit* habitat" OR "limit* area*" OR "restrict*distribution" OR "restrict* range" OR "restrict* extent" OR "restrict* niche" OR "restrict* habitat" OR "restrict* area*" OR "confin* distribution" OR "confin* range" OR "confin* extent" OR "confin* niche" OR "confin* habitat" OR "confin* area*" OR "declin* distribution" OR "declin* range" OR "declin* extent" OR "declin* habitat" OR "declin* niche" OR "declin* habitat" OR "declin* area" OR "decreas* distribution" OR "decreas* range" OR "decreas* extent" OR "decreas* habitat" OR "decreas* niche" OR "decreas* habitat" OR "decreas* area" OR ("loss " AND "distribution") OR ("loss " AND "range ") OR ("loss " AND "extent") OR ("loss " AND "niche") OR ("loss" AND "habitat"))**

AND TITLE-ABS-KEY (“pest regulat*” OR “disease regulat*” OR “pollin*” OR “nutrient cycl*” OR “soil form*” OR “climate regulat*” OR “natural-hazard regulat*” OR “water cycl*” OR “habitat provis*”)

AND TITLE-ABS-KEY (“farm*” OR “agri*” OR “food”)

AND TITLE-ABS-KEY ("contribut*" OR "produc*" OR "return*" OR "profit*" OR revenue OR return OR output OR "incom*")

AND (EXCLUDE (PUBYEAR, 2022)) AND (LIMIT-TO (LANGUAGE, “English”))

AND NOT (aquatic OR marine OR livestock OR cattle OR pig OR bushmeat OR “wetland*” OR “peatland*” OR “medicin*”)

**- 303 documents**

TITLE-ABS-KEY (**"loss " AND "area")**

AND TITLE-ABS-KEY (“ecosystem servic*” OR “ecosystem function*”)

AND TITLE-ABS-KEY (“farm*” OR “agri*” OR “food”)

AND TITLE-ABS-KEY ("contribut*" OR "produc*" OR "return*" OR "profit*" OR revenue OR return OR output OR "incom*")

AND (EXCLUDE (PUBYEAR, 2022)) AND (LIMIT-TO (LANGUAGE, “English”))

AND NOT (aquatic OR marine OR livestock OR cattle OR pig OR bushmeat OR “wetland*” OR “peatland*” OR “medicin*”)

**- 182 documents**

TITLE-ABS-KEY (**"loss " AND "area")**

AND TITLE-ABS-KEY (“pest regulat*” OR “disease regulat*” OR “pollin*” OR “nutrient cycl*” OR “soil form*” OR “climate regulat*” OR “natural-hazard regulat*” OR “water cycl*” OR “habitat provis*”)

AND TITLE-ABS-KEY (“farm*” OR “agri*” OR “food”)

AND TITLE-ABS-KEY ("contribut*" OR "produc*" OR "return*" OR "profit*" OR revenue OR return OR output OR "incom*")

AND (EXCLUDE (PUBYEAR, 2022)) AND (LIMIT-TO (LANGUAGE, “English”))

AND NOT (aquatic OR marine OR livestock OR cattle OR pig OR bushmeat OR “wetland*” OR “peatland*” OR “medicin*”)

#### Unit measure contribution

**- 148 documents**

TITLE-ABS-KEY ("small distribution" OR "small range" OR "small extent" OR "small niche" OR "small habitat" OR "small area" OR "limit*distribution" OR "limit* range" OR "limit* extent" OR "limit* niche" OR "limit* habitat" OR "limit* area*" OR "restrict*distribution" OR "restrict* range" OR "restrict* extent" OR "restrict* niche" OR "restrict* habitat" OR "restrict* area*" OR "confin* distribution" OR "confin* range" OR "confin* extent" OR "confin* niche" OR "confin* habitat" OR "confin* area*" OR "declin* distribution" OR "declin* range" OR "declin* extent" OR "declin* habitat" OR "declin* niche" OR "declin* habitat" OR "declin* area" OR "decreas* distribution" OR "decreas* range" OR "decreas* extent" OR "decreas* habitat" OR "decreas* niche" OR "decreas* habitat" OR "decreas* area" OR ("loss " AND "distribution") OR ("loss " AND "range ") OR ("loss " AND "extent") OR ("loss " AND "niche") OR ("loss" AND "habitat") OR ("loss " AND "area"))

AND TITLE-ABS-KEY (“ecosystem servic*” OR “ecosystem function*” OR “pest regulat*” OR “disease regulat*” OR “pollin*” OR “nutrient cycl*” OR “soil form*” OR “climate regulat*” OR “natural-hazard regulat*” OR “water cycl*” OR “habitat provis*”)

AND TITLE-ABS-KEY (“farm*” OR “agri*” OR “food”)

AND TITLE-ABS-KEY (kg OR tonnes OR "$" OR "dollar*" OR kcal OR calories OR hectare OR "ha" OR “ton*” OR “bushel*”)

AND (EXCLUDE (PUBYEAR, 2022)) AND (LIMIT-TO (LANGUAGE, “English”))

AND NOT (aquatic OR marine OR livestock OR cattle OR pig OR bushmeat OR “wetland*” OR “peatland*” OR “medicin*”)

#### Food contribution

**- 255 documents**

TITLE-ABS-KEY ("small distribution" OR "small range" OR "small extent" OR "small niche" OR "small habitat" OR "small area" OR "limit*distribution" OR "limit* range" OR "limit* extent" OR "limit* niche" OR "limit* habitat" OR "limit* area*" OR "restrict*distribution" OR "restrict* range" OR "restrict* extent" OR "restrict* niche" OR "restrict* habitat" OR "restrict* area*" OR "confin* distribution" OR "confin* range" OR "confin* extent" OR "confin* niche" OR "confin* habitat" OR "confin* area*" OR "declin* distribution" OR "declin* range" OR "declin* extent" OR "declin* habitat" OR "declin* niche" OR "declin* habitat" OR "declin* area" OR "decreas* distribution" OR "decreas* range" OR "decreas* extent" OR "decreas* habitat" OR "decreas* niche" OR "decreas* habitat" OR "decreas* area" OR ("loss " AND "distribution") OR ("loss " AND "range ") OR ("loss " AND "extent") OR ("loss " AND "niche") OR ("loss" AND "habitat") OR ("loss " AND "area"))

AND TITLE-ABS-KEY (“ecosystem servic*” OR “ecosystem function*”)

AND TITLE-ABS-KEY (“farm*” OR “agri*” OR “food”)

AND TITLE-ABS-KEY (crop OR "harvest*" OR yield)

AND (EXCLUDE (PUBYEAR, 2022)) AND (LIMIT-TO (LANGUAGE, “English”))

AND NOT (aquatic OR marine OR livestock OR cattle OR pig OR bushmeat OR “wetland*” OR “peatland*” OR “medicin*”)

**- 273 documents**

TITLE-ABS-KEY ("small distribution" OR "small range" OR "small extent" OR "small niche" OR "small habitat" OR "small area" OR "limit*distribution" OR "limit* range" OR "limit* extent" OR "limit* niche" OR "limit* habitat" OR "limit* area*" OR "restrict*distribution" OR "restrict* range" OR "restrict* extent" OR "restrict* niche" OR "restrict* habitat" OR "restrict* area*" OR "confin*distribution" OR "confin* range" OR "confin* extent" OR "confin* niche" OR "confin* habitat" OR "confin* area*" OR "declin* distribution" OR "declin* range" OR "declin* extent" OR "declin* habitat" OR "declin* niche" OR "declin* habitat" OR "declin* area" OR "decreas* distribution" OR "decreas* range" OR "decreas* extent" OR "decreas* habitat" OR "decreas* niche" OR "decreas* habitat" OR "decreas* area" OR ("loss " AND "distribution") OR ("loss " AND "range ") OR ("loss " AND "extent") OR ("loss " AND "niche") OR ("loss" AND "habitat") OR ("loss " AND "area"))

AND TITLE-ABS-KEY (“pest regulat*” OR “disease regulat*” OR “pollin*” OR “nutrient cycl*” OR “soil form*” OR “climate regulat*” OR “natural-hazard regulat*” OR “water cycl*” OR “habitat provis*”)

AND TITLE-ABS-KEY (“farm*” OR “agri*” OR “food”)

AND TITLE-ABS-KEY (crop OR "harvest*" OR yield)

AND (EXCLUDE (PUBYEAR, 2022)) AND (LIMIT-TO (LANGUAGE, “English”))

AND NOT (aquatic OR marine OR livestock OR cattle OR pig OR bushmeat OR “wetland*” OR “peatland*” OR “medicin*”)

### ABUNDANCE

#### General contribution

**- 232 documents**

**TITLE-ABS-KEY ("low abundance" OR "low number*" OR "low population" OR “low density” OR "small abundance" OR "small numbers" OR "small population" OR "few individuals" OR "few populations" OR "limit* abundance" OR "limit* number*" OR "limit* population*" OR "restrict* abundance" OR "restrict* number*" OR "restrict* population*" OR "confin* abundance" OR "confin* number*" OR "confin* population*" OR "declin* abundance" OR "declin* number*" OR "declin* population*" OR "decreas* abundance" OR "decreas* number*" OR "decreas* population*" OR "fall* abundance" OR "fall* number*" OR ("loss " AND "abundance") OR ("loss " AND "number*") OR ("loss " AND "individual*"))**

AND TITLE-ABS-KEY (“ecosystem servic*” OR “ecosystem function*”)

AND TITLE-ABS-KEY (“farm*” OR “agri*” OR “food”)

AND TITLE-ABS-KEY ("contribut*" OR "produc*" OR "return*" OR "profit*" OR revenue OR return OR output OR "incom*")

AND (EXCLUDE (PUBYEAR, 2022)) AND (LIMIT-TO (LANGUAGE, “English”))

AND NOT (aquatic OR marine OR livestock OR cattle OR pig OR bushmeat OR “wetland*” OR “peatland*” OR “medicin*”)

- **168** **documents**

TITLE-ABS-**KEY ("loss " AND "population*")**

AND TITLE-ABS-KEY (“ecosystem servic*” OR “ecosystem function*”)

AND TITLE-ABS-KEY (“farm*” OR “agri*” OR “food”)

AND TITLE-ABS-KEY ("contribut*" OR "produc*" OR "return*" OR "profit*" OR revenue OR return OR output OR "incom*")

AND (EXCLUDE (PUBYEAR, 2022)) AND (LIMIT-TO (LANGUAGE, “English”))

AND NOT (aquatic OR marine OR livestock OR cattle OR pig OR bushmeat OR “wetland*” OR “peatland*” OR “medicin*”)

**- 292 documents**

**TITLE-ABS-KEY ("low abundance" OR "low number*" OR "low population" OR “low density” OR "small abundance" OR "small numbers" OR "small population" OR "few individuals" OR "few populations" OR "limit* abundance" OR "limit* number*" OR "limit* population*" OR "restrict* abundance" OR "restrict* number*" OR "restrict* population*" OR "confin* abundance" OR "confin* number*" OR "confin* population*" OR "declin* abundance" OR "declin* number*" OR "declin* population*" OR "decreas* abundance" OR "decreas* number*" OR "decreas* population*" OR "fall* abundance" OR "fall* number*" OR ("loss " AND "abundance") OR ("loss " AND "number*") OR ("loss " AND "individual*"))**

AND TITLE-ABS-KEY (“pest regulat*” OR “disease regulat*” OR “pollin*” OR “nutrient cycl*” OR “soil form*” OR “climate regulat*” OR “natural-hazard regulat*” OR “water cycl*” OR “habitat provis*”)

AND TITLE-ABS-KEY (“farm*” OR “agri*” OR “food”)

AND TITLE-ABS-KEY ("contribut*" OR "produc*" OR "return*" OR "profit*" OR revenue OR return OR output OR "incom*")

AND (EXCLUDE (PUBYEAR, 2022)) AND (LIMIT-TO (LANGUAGE, “English”))

AND NOT (aquatic OR marine OR livestock OR cattle OR pig OR bushmeat OR “wetland*” OR “peatland*” OR “medicin*”)

- **177 documents**

TITLE-ABS-**KEY ("loss " AND "population*")**

AND TITLE-ABS-KEY (“pest regulat*” OR “disease regulat*” OR “pollin*” OR “nutrient cycl*” OR “soil form*” OR “climate regulat*” OR “natural-hazard regulat*” OR “water cycl*” OR “habitat provis*”)

AND TITLE-ABS-KEY (“farm*” OR “agri*” OR “food”)

AND TITLE-ABS-KEY ("contribut*" OR "produc*" OR "return*" OR "profit*" OR revenue OR return OR output OR "incom*")

AND (EXCLUDE (PUBYEAR, 2022)) AND (LIMIT-TO (LANGUAGE, “English”))

AND NOT (aquatic OR marine OR livestock OR cattle OR pig OR bushmeat OR “wetland*” OR “peatland*” OR “medicin*”)

#### Unit measure contribution

**- 106 documents**

TITLE-ABS-KEY ("low abundance" OR "low number*" OR "low population" OR “low density” OR "small abundance" OR "small numbers" OR "small population" OR "few individuals" OR "few populations" OR "limit* abundance" OR "limit* number*" OR "limit*population*" OR "restrict* abundance" OR "restrict* number*" OR "restrict* population*" OR "confin* abundance" OR "confin* number*" OR "confin* population*" OR "declin* abundance" OR "declin* number*" OR "declin* population*" OR "decreas* abundance" OR "decreas* number*" OR "decreas* population*" OR "fall* abundance" OR "fall* number*" OR ("loss " AND "abundance") OR ("loss " AND "number*") OR ("loss " AND "individual*") OR ("loss " AND "population*"))

AND TITLE-ABS-KEY (“ecosystem servic*” OR “ecosystem function*” OR “pest regulat*” OR “disease regulat*” OR “pollin*” OR “nutrient cycl*” OR “soil form*” OR “climate regulat*” OR “natural-hazard regulat*” OR “water cycl*” OR “habitat provis*”)

AND TITLE-ABS-KEY (“farm*” OR “agri*” OR “food”)

AND TITLE-ABS-KEY (kg OR tonnes OR "$" OR "dollar*" OR kcal OR calories OR hectare OR "ha" OR “ton*” OR “bushel*”)

AND (EXCLUDE (PUBYEAR, 2022)) AND (LIMIT-TO (LANGUAGE, “English”))

AND NOT (aquatic OR marine OR livestock OR cattle OR pig OR bushmeat OR “wetland*” OR “peatland*” OR “medicin*”)

#### Food contribution

**- 224 documents**

TITLE-ABS-KEY ("low abundance" OR "low number*" OR "low population" OR “low density” OR "small abundance" OR "small numbers" OR "small population" OR "few individuals" OR "few populations" OR "limit* abundance" OR "limit* number*" OR "limit*population*" OR "restrict* abundance" OR "restrict* number*" OR "restrict* population*" OR "confin* abundance" OR "confin* number*" OR "confin* population*" OR "declin* abundance" OR "declin* number*" OR "declin* population*" OR "decreas* abundance" OR "decreas* number*" OR "decreas* population*" OR "fall* abundance" OR "fall* number*" OR ("loss " AND "abundance") OR ("loss " AND "number*") OR ("loss " AND "individual*") OR ("loss " AND "population*"))

AND TITLE-ABS-KEY (“ecosystem servic*” OR “ecosystem function*”)

AND TITLE-ABS-KEY (“farm*” OR “agri*” OR “food”)

AND TITLE-ABS-KEY (crop OR "harvest*" OR yield)

AND (EXCLUDE (PUBYEAR, 2022)) AND (LIMIT-TO (LANGUAGE, “English”))

AND NOT (aquatic OR marine OR livestock OR cattle OR pig OR bushmeat OR “wetland*” OR “peatland*” OR “medicin*”)

**- 340 documents**

TITLE-ABS-KEY ("low abundance" OR "low number*" OR "low population" OR “low density” OR "small abundance" OR "small numbers" OR "small population" OR "few individuals" OR "few populations" OR "limit* abundance" OR "limit* number*" OR "limit*population*" OR "restrict* abundance" OR "restrict* number*" OR "restrict*population*" OR "confin* abundance" OR "confin* number*" OR "confin* population*" OR "declin* abundance" OR "declin* number*" OR "declin* population*" OR "decreas* abundance" OR "decreas* number*" OR "decreas* population*" OR "fall* abundance" OR "fall* number*" OR ("loss " AND "abundance") OR ("loss " AND "number*") OR ("loss " AND "individual*") OR ("loss " AND "population*"))

AND TITLE-ABS-KEY (“pest regulat*” OR “disease regulat*” OR “pollin*” OR “nutrient cycl*” OR “soil form*” OR “climate regulat*” OR “natural-hazard regulat*” OR “water cycl*” OR “habitat provis*”)

AND TITLE-ABS-KEY (“farm*” OR “agri*” OR “food”)

AND TITLE-ABS-KEY (crop OR "harvest*" OR yield)

AND (EXCLUDE (PUBYEAR, 2022)) AND (LIMIT-TO (LANGUAGE, "English"))

AND NOT (aquatic OR marine OR livestock OR cattle OR pig OR bushmeat OR “wetland*” OR “peatland*” OR “medicin*”)

### European Red List of bees (Nieto et al., 2014)

#### General contribution

**- 1 document**

TITLE-ABS-KEY (“Ammobates dusmeti” OR “Andrena labiatula” OR “Andrena ornata” OR “Andrena tridentata” OR “Bombus cullumanus” OR “Megachile cypricola” OR “Nomada siciliensis” OR “Ammobates electoides” OR “Ammobatoides abdominalis” OR “Andrena comta” OR “Colletes wolfi” OR “Dasypoda braccata” OR “Andrena magna” OR “Andrena stepposa” OR “Andrena stigmatica ” OR “Bombus armeniacus” OR “Bombus brodmannicus” OR “Bombus fragrans” OR “Bombus inexspectatus” OR “Bombus mocsaryi” OR “Bombus reinigiellus” OR “Bombus zonatus” OR “Colletes anchusae” OR “Colletes caspicus” OR “Colletes collaris” OR “Colletes graeffei” OR “Colletes merceti” OR “Colletes meyeri” OR “Colletes punctatus” OR “Colletes sierrensis” OR “Dasypoda frieseana” OR “Dasypoda spinigera” OR “Dasypoda suripes” OR “Flavipanurgus granadensis” OR “Halictus arinthiacus” OR “Halictus microcardia” OR “Halictus semitectus” OR “Icteranthidium cimbiciforme” OR “Lasioglossum breviventre” OR “Lasioglossum laeve” OR “Lasioglossum quadrisig natum” OR “Lasioglossum sexmaculatum” OR “Lasioglossum sexnotatulum” OR “Lasioglossum soror” OR “Lasioglossum subfasciatum” OR “Lasioglossum virens” OR “Melitta melanura” OR “Nomada italica” OR “Nomada pulchra” OR “Osmia maritima” OR “Parammobatodes minutus” OR “Trachusa interrupta” OR “Andrena transitoria” OR “Biastes truncatus” OR “Bombus alpinus” OR “Bombus confusus” OR “Bombus distinguendus” OR “Bombus gerstaeckeri” OR “Bombus hyperboreus” OR “Bombus muscorum” OR “Bombus polaris” OR “Bombus pomorum” OR “Coelioxys elongatula” OR “Colletes chengtehensis” OR “Colletes dimidiatus” OR “Colletes floralis” OR “Colletes fodiens” OR “Colletes impunctatus” OR “Colletes moricei” OR “Colletes perezi” OR “Colletes pulchellus” OR “Halictus leucaheneus” OR “Melitta hispanica” OR “Melitta kastiliensis” OR “Nomada noskiewiczi” OR “Systropha planidens” OR “Andrena nanaeformis”)

AND TITLE-ABS-KEY (“ecosystem servic*” OR “ecosystem funct*” OR “pest regulat*” OR “disease regulat*” OR “pollin*” OR “nutrient cycl*” OR “soil form*” OR “climate regulat*” OR “natural-hazard regulat*” OR “water cycl*” OR “habitat provis*”)

AND TITLE-ABS-KEY (“farm*” OR “agri*” OR “food”)

AND TITLE-ABS-KEY ("contribut*" OR "produc*" OR "return*" OR "profit*" OR revenue OR return OR output OR "incom*")

AND (EXCLUDE (PUBYEAR, 2022)) AND (LIMIT-TO (LANGUAGE, "English"))

AND NOT (aquatic OR marine OR livestock OR cattle OR pig OR bushmeat OR “wetland*” OR “peatland*” OR “medicin*”)

#### Unit measure contribution

**- 2 documents**

TITLE-ABS-KEY (“Ammobates dusmeti” OR “Andrena labiatula” OR “Andrena ornata” OR “Andrena tridentata” OR “Bombus cullumanus” OR “Megachile cypricola” OR “Nomada siciliensis” OR “Ammobates electoides” OR “Ammobatoides abdominalis” OR “Andrena comta” OR “Colletes wolfi” OR “Dasypoda braccata” OR “Andrena magna” OR “Andrena stepposa” OR “Andrena stigmatica ” OR “Bombus armeniacus” OR “Bombus brodmannicus” OR “Bombus fragrans” OR “Bombus inexspectatus” OR “Bombus mocsaryi” OR “Bombus reinigiellus” OR “Bombus zonatus” OR “Colletes anchusae” OR “Colletes caspicus” OR “Colletes collaris” OR “Colletes graeffei” OR “Colletes merceti” OR “Colletes meyeri” OR “Colletes punctatus” OR “Colletes sierrensis” OR “Dasypoda frieseana” OR “Dasypoda spinigera” OR “Dasypoda suripes” OR “Flavipanurgus granadensis” OR “Halictus arinthiacus” OR “Halictus microcardia” OR “Halictus semitectus” OR “Icteranthidium cimbiciforme” OR “Lasioglossum breviventre” OR “Lasioglossum laeve” OR “Lasioglossum quadrisig natum” OR “Lasioglossum sexmaculatum” OR “Lasioglossum sexnotatulum” OR “Lasioglossum soror” OR “Lasioglossum subfasciatum” OR “Lasioglossum virens” OR “Melitta melanura” OR “Nomada italica” OR “Nomada pulchra” OR “Osmia maritima” OR “Parammobatodes minutus” OR “Trachusa interrupta” OR “Andrena transitoria” OR “Biastes truncatus” OR “Bombus alpinus” OR “Bombus confusus” OR “Bombus distinguendus” OR “Bombus gerstaeckeri” OR “Bombus hyperboreus” OR “Bombus muscorum” OR “Bombus polaris” OR “Bombus pomorum” OR “Coelioxys elongatula” OR “Colletes chengtehensis” OR “Colletes dimidiatus” OR “Colletes floralis” OR “Colletes fodiens” OR “Colletes impunctatus” OR “Colletes moricei” OR “Colletes perezi” OR “Colletes pulchellus” OR “Halictus leucaheneus” OR “Melitta hispanica” OR “Melitta kastiliensis” OR “Nomada noskiewiczi” OR “Systropha planidens” OR “Andrena nanaeformis”)

AND TITLE-ABS-KEY (“ecosystem servic*” OR “ecosystem function*” OR “pest regulat*” OR “disease regulat*” OR “pollin*” OR “nutrient cycl*” OR “soil form*” OR “climate regulat*” OR “natural-hazard regulat*” OR “water cycl*” OR “habitat provis*”)

AND TITLE-ABS-KEY (“farm*” OR “agri*” OR “food”)

AND TITLE-ABS-KEY (kg OR tonnes OR "$" OR "dollar*" OR kcal OR calories OR hectare OR "ha" OR “ton*” OR “bushel*”)

AND (EXCLUDE (PUBYEAR, 2022)) AND (LIMIT-TO (LANGUAGE, “English”))

AND NOT (aquatic OR marine OR livestock OR cattle OR pig OR bushmeat OR “wetland*” OR “peatland*” OR “medicin*”)

#### Food contribution

**- 1 document**

TITLE-ABS-KEY (“Ammobates dusmeti” OR “Andrena labiatula” OR “Andrena ornata” OR “Andrena tridentata” OR “Bombus cullumanus” OR “Megachile cypricola” OR “Nomada siciliensis” OR “Ammobates electoides” OR “Ammobatoides abdominalis” OR “Andrena comta” OR “Colletes wolfi” OR “Dasypoda braccata” OR “Andrena magna” OR “Andrena stepposa” OR “Andrena stigmatica ” OR “Bombus armeniacus” OR “Bombus brodmannicus” OR “Bombus fragrans” OR “Bombus inexspectatus” OR “Bombus mocsaryi” OR “Bombus reinigiellus” OR “Bombus zonatus” OR “Colletes anchusae” OR “Colletes caspicus” OR “Colletes collaris” OR “Colletes graeffei” OR “Colletes merceti” OR “Colletes meyeri” OR “Colletes punctatus” OR “Colletes sierrensis” OR “Dasypoda frieseana” OR “Dasypoda spinigera” OR “Dasypoda suripes” OR “Flavipanurgus granadensis” OR “Halictus arinthiacus” OR “Halictus microcardia” OR “Halictus semitectus” OR “Icteranthidium cimbiciforme” OR “Lasioglossum breviventre” OR “Lasioglossum laeve” OR “Lasioglossum quadrisig natum” OR “Lasioglossum sexmaculatum” OR “Lasioglossum sexnotatulum” OR “Lasioglossum soror” OR “Lasioglossum subfasciatum” OR “Lasioglossum virens” OR “Melitta melanura” OR “Nomada italica” OR “Nomada pulchra” OR “Osmia maritima” OR “Parammobatodes minutus” OR “Trachusa interrupta” OR “Andrena transitoria” OR “Biastes truncatus” OR “Bombus alpinus” OR “Bombus confusus” OR “Bombus distinguendus” OR “Bombus gerstaeckeri” OR “Bombus hyperboreus” OR “Bombus muscorum” OR “Bombus polaris” OR “Bombus pomorum” OR “Coelioxys elongatula” OR “Colletes chengtehensis” OR “Colletes dimidiatus” OR “Colletes floralis” OR “Colletes fodiens” OR “Colletes impunctatus” OR “Colletes moricei” OR “Colletes perezi” OR “Colletes pulchellus” OR “Halictus leucaheneus” OR “Melitta hispanica” OR “Melitta kastiliensis” OR “Nomada noskiewiczi” OR “Systropha planidens” OR “Andrena nanaeformis”)

AND TITLE-ABS-KEY (“ecosystem servic*” OR “ecosystem function*” OR “pest regulat*” OR “disease regulat*” OR “pollin*” OR “nutrient cycl*” OR “soil form*” OR “climate regulat*” OR “natural-hazard regulat*” OR “water cycl*” OR “habitat provis*”)

AND TITLE-ABS-KEY (“farm*” OR “agri*” OR “food”)

AND TITLE-ABS-KEY (crop OR "harvest*" OR yield)

AND (EXCLUDE (PUBYEAR, 2022)) AND (LIMIT-TO (LANGUAGE, “English”))

AND NOT (aquatic OR marine OR livestock OR cattle OR pig OR bushmeat OR “wetland*” OR “peatland*” OR “medicin*”)

### Red listed pollinating insects of North America (National Research Council, 2007)

#### General contribution

**- 3 documents**

TITLE-ABS-KEY (“Agathymus evansi” OR “Amblyscirtes linda” OR “Apodemia mormo langei” OR “Atrytone arogos” OR “Boloria acrocnema” OR “Boloria alberta” OR “Calephelis borealis” OR “Callophrys comstocki” OR “Callophrys irus” OR “Callophrys lanoraieensis” OR “Callophrys mossii bayensis” OR “Celotes limpia” OR “Cyclargus thomasi bethunebakeri” OR “Erora laeta” OR “Erynnis persius persius” OR “Euchloe ausonides insulana” OR “Euphilotes battoides allyni” OR “Euphilotes baueri” OR “Euphilotes enoptes smithi” OR “Euphilotes mojave” OR “Euphydryas anicia cloudcrofti” OR “Euphydryas editha bayensis” OR “Euphydryas editha quino” OR “Euphydryas editha taylori” OR “Euphydryas gillettii” OR “Euphyes bayensis” OR “Euphyes dukesi” OR “Euproserpinus Euterpe” OR “Fixsenia [Satyrium] polingi” OR “Glaucopsyche lygdamus palosverdesensis” OR “Heraclides aristodemus ponceanus” OR “Hesperia dacotae” OR “Hesperia leonardus montana” OR “Hesperia ottoe” OR “Hesperopsis gracielae” OR “Icaricia icarioides fender” OR “Icaricia icarioides missionensis” OR “Lycaeides idas lotis” OR “Lycaeides melissa samuelis” OR “Manduca blackburni” OR “Mitoura hesseli” OR “Neonympha mitchellii francisci” OR “Neonympha mitchellii mitchellii” OR “Oarisma powesheik” OR “Papilio joanae” OR “Polites mardon” OR “Problema bulenta” OR “Problema byssus” OR “Pseudocopaeodes eunus obscurus” OR “Pyrgus ruralis lagunae” OR “Satyrium kingi” OR “Speyeria callippe callippe” OR “Speyeria diana” OR “Speyeria idalia” OR “Speyeria zerene behrensii” OR “Speyeria zerene Hippolyta” OR “Speyeria zerene myrtleae” OR “Stallingsia maculosus” OR “Andrena aculeata” OR “Andrena winnemuccana” OR “Ashmeadiella sculleni” OR “Bombus affinis” OR “Bombus franklini” OR “Bombus lucorum” OR “Bombus occidentalis” OR “Bombus terricola” OR “Calliopsis barri” OR “Epeoloides pilosula” OR “Eucera douglasiana” OR “Eucera frater lata” OR “Halictus harmonius“ OR “Halictus pinguismentus” OR “Hesperapis kayella” OR “Hoplitis orthognathus” OR “Hoplitis producta subgracilis” OR “Hylaeus akoko” OR “Hylaeus anomalus” OR “Hylaeus anthracinus” OR “Hylaeus assimulans” OR “Hylaeus dimidiatus” OR “Hylaeus facilis” OR “Hylaeus finitimus” OR “Hylaeus flavifrons” OR “Hylaeus gliddenae” OR “Hylaeus hilaris” OR “Hylaeus hula” OR “Hylaeus kona” OR “Hylaeus kuakea” OR “Hylaeus longiceps” OR “Hylaeus lunicraterius” OR “Hylaeus mana” OR “Hylaeus mauiensis” OR “Hylaeus melanothrix” OR “Hylaeus nalo” OR “Hylaeus niloticus” OR “Hylaeus ombrias” OR “Hylaeus paradoxicus” OR “Hylaeus perspicuous” OR “Hylaeus psammobius” OR “Hylaeus satelles” OR “Hylaeus simplex” OR “Hylaeus solaris” OR “Macropis steironema opaca” OR “Osmia ashmeadii” OR “Osmia cascadica” OR “Perdita salicis euxantha” OR “Perdita salici sublaeta” OR “Perdita similes Pascoensis” OR “Perdita wyomingensis Sculleni” OR “Protandrena subdilatipes” OR “Sphecodogastra antiochensis”)

AND TITLE-ABS-KEY (“ecosystem servic*” OR “ecosystem funct*” OR “pest regulat*” OR “disease regulat*” OR “pollin*” OR “nutrient cycl*” OR “soil form*” OR “climate regulat*” OR “natural-hazard regulat*” OR “water cycl*” OR “habitat provis*”)

AND TITLE-ABS-KEY (“farm*” OR “agri*” OR “food”)

AND TITLE-ABS-KEY ("contribut*" OR "produc*" OR "return*" OR "profit*" OR revenue OR return OR output OR "incom*")

AND (EXCLUDE (PUBYEAR, 2022)) AND (LIMIT-TO (LANGUAGE, “English”))

AND NOT (aquatic OR marine OR livestock OR cattle OR pig OR bushmeat OR “wetland*” OR “peatland*” OR “medicin*”)

#### Unit measure contribution

**- 0 documents**

TITLE-ABS-KEY (“Agathymus evansi” OR “Amblyscirtes linda” OR “Apodemia mormo langei” OR “Atrytone arogos” OR “Boloria acrocnema” OR “Boloria alberta” OR “Calephelis borealis” OR “Callophrys comstocki” OR “Callophrys irus” OR “Callophrys lanoraieensis” OR “Callophrys mossii bayensis” OR “Celotes limpia” OR “Cyclargus thomasi bethunebakeri” OR “Erora laeta” OR “Erynnis persius persius” OR “Euchloe ausonides insulana” OR “Euphilotes battoides allyni” OR “Euphilotes baueri” OR “Euphilotes enoptes smithi” OR “Euphilotes mojave” OR “Euphydryas anicia cloudcrofti” OR “Euphydryas editha bayensis” OR “Euphydryas editha quino” OR “Euphydryas editha taylori” OR “Euphydryas gillettii” OR “Euphyes bayensis” OR “Euphyes dukesi” OR “Euproserpinus Euterpe” OR “Fixsenia [Satyrium] polingi” OR “Glaucopsyche lygdamus palosverdesensis” OR “Heraclides aristodemus ponceanus” OR “Hesperia dacotae” OR “Hesperia leonardus montana” OR “Hesperia ottoe” OR “Hesperopsis gracielae” OR “Icaricia icarioides fender” OR “Icaricia icarioides missionensis” OR “Lycaeides idas lotis” OR “Lycaeides melissa samuelis” OR “Manduca blackburni” OR “Mitoura hesseli” OR “Neonympha mitchellii francisci” OR “Neonympha mitchellii mitchellii” OR “Oarisma powesheik” OR “Papilio joanae” OR “Polites mardon” OR “Problema bulenta” OR “Problema byssus” OR “Pseudocopaeodes eunus obscurus” OR “Pyrgus ruralis lagunae” OR “Satyrium kingi” OR “Speyeria callippe callippe” OR “Speyeria diana” OR “Speyeria idalia” OR “Speyeria zerene behrensii” OR “Speyeria zerene Hippolyta” OR “Speyeria zerene myrtleae” OR “Stallingsia maculosus” OR “Andrena aculeata” OR “Andrena winnemuccana” OR “Ashmeadiella sculleni” OR “Bombus affinis” OR “Bombus franklini” OR “Bombus lucorum” OR “Bombus occidentalis” OR “Bombus terricola” OR “Calliopsis barri” OR “Epeoloides pilosula” OR “Eucera douglasiana” OR “Eucera frater lata” OR “Halictus harmonius“ OR “Halictus pinguismentus” OR “Hesperapis kayella” OR “Hoplitis orthognathus” OR “Hoplitis producta subgracilis” OR “Hylaeus akoko” OR “Hylaeus anomalus” OR “Hylaeus anthracinus” OR “Hylaeus assimulans” OR “Hylaeus dimidiatus” OR “Hylaeus facilis” OR “Hylaeus finitimus” OR “Hylaeus flavifrons” OR “Hylaeus gliddenae” OR “Hylaeus hilaris” OR “Hylaeus hula” OR “Hylaeus kona” OR “Hylaeus kuakea” OR “Hylaeus longiceps” OR “Hylaeus lunicraterius” OR “Hylaeus mana” OR “Hylaeus mauiensis” OR “Hylaeus melanothrix” OR “Hylaeus nalo” OR “Hylaeus niloticus” OR “Hylaeus ombrias” OR “Hylaeus paradoxicus” OR “Hylaeus perspicuous” OR “Hylaeus psammobius” OR “Hylaeus satelles” OR “Hylaeus simplex” OR “Hylaeus solaris” OR “Macropis steironema opaca” OR “Osmia ashmeadii” OR “Osmia cascadica” OR “Perdita salicis euxantha” OR “Perdita salici sublaeta” OR “Perdita similes Pascoensis” OR “Perdita wyomingensis Sculleni” OR “Protandrena subdilatipes” OR “Sphecodogastra antiochensis”)

AND TITLE-ABS-KEY (“ecosystem servic*” OR “ecosystem function*” OR “pest regulat*” OR “disease regulat*” OR “pollin*” OR “nutrient cycl*” OR “soil form*” OR “climate regulat*” OR “natural-hazard regulat*” OR “water cycl*” OR “habitat provis*”)

AND TITLE-ABS-KEY (“farm*” OR “agri*” OR “food”)

AND TITLE-ABS-KEY (kg OR tonnes OR "$" OR "dollar*" OR kcal OR calories OR hectare OR "ha" OR “ton*” OR “bushel*”)

AND (EXCLUDE (PUBYEAR, 2022)) AND (LIMIT-TO (LANGUAGE, “English”))

AND NOT (aquatic OR marine OR livestock OR cattle OR pig OR bushmeat OR “wetland*” OR “peatland*” OR “medicin*”)

#### Food contribution

**- 6 documents**

TITLE-ABS-KEY (“Agathymus evansi” OR “Amblyscirtes linda” OR “Apodemia mormo langei” OR “Atrytone arogos” OR “Boloria acrocnema” OR “Boloria alberta” OR “Calephelis borealis” OR “Callophrys comstocki” OR “Callophrys irus” OR “Callophrys lanoraieensis” OR “Callophrys mossii bayensis” OR “Celotes limpia” OR “Cyclargus thomasi bethunebakeri” OR “Erora laeta” OR “Erynnis persius persius” OR “Euchloe ausonides insulana” OR “Euphilotes battoides allyni” OR “Euphilotes baueri” OR “Euphilotes enoptes smithi” OR “Euphilotes mojave” OR “Euphydryas anicia cloudcrofti” OR “Euphydryas editha bayensis” OR “Euphydryas editha quino” OR “Euphydryas editha taylori” OR “Euphydryas gillettii” OR “Euphyes bayensis” OR “Euphyes dukesi” OR “Euproserpinus Euterpe” OR “Fixsenia [Satyrium] polingi” OR “Glaucopsyche lygdamus palosverdesensis” OR “Heraclides aristodemus ponceanus” OR “Hesperia dacotae” OR “Hesperia leonardus montana” OR “Hesperia ottoe” OR “Hesperopsis gracielae” OR “Icaricia icarioides fender” OR “Icaricia icarioides missionensis” OR “Lycaeides idas lotis” OR “Lycaeides melissa samuelis” OR “Manduca blackburni” OR “Mitoura hesseli” OR “Neonympha mitchellii francisci” OR “Neonympha mitchellii mitchellii” OR “Oarisma powesheik” OR “Papilio joanae” OR “Polites mardon” OR “Problema bulenta” OR “Problema byssus” OR “Pseudocopaeodes eunus obscurus” OR “Pyrgus ruralis lagunae” OR “Satyrium kingi” OR “Speyeria callippe callippe” OR “Speyeria diana” OR “Speyeria idalia” OR “Speyeria zerene behrensii” OR “Speyeria zerene Hippolyta” OR “Speyeria zerene myrtleae” OR “Stallingsia maculosus” OR “Andrena aculeata” OR “Andrena winnemuccana” OR “Ashmeadiella sculleni” OR “Bombus affinis” OR “Bombus franklini” OR “Bombus lucorum” OR “Bombus occidentalis” OR “Bombus terricola” OR “Calliopsis barri” OR “Epeoloides pilosula” OR “Eucera douglasiana” OR “Eucera frater lata” OR “Halictus harmonius“ OR “Halictus pinguismentus” OR “Hesperapis kayella” OR “Hoplitis orthognathus” OR “Hoplitis producta subgracilis” OR “Hylaeus akoko” OR “Hylaeus anomalus” OR “Hylaeus anthracinus” OR “Hylaeus assimulans” OR “Hylaeus dimidiatus” OR “Hylaeus facilis” OR “Hylaeus finitimus” OR “Hylaeus flavifrons” OR “Hylaeus gliddenae” OR “Hylaeus hilaris” OR “Hylaeus hula” OR “Hylaeus kona” OR “Hylaeus kuakea” OR “Hylaeus longiceps” OR “Hylaeus lunicraterius” OR “Hylaeus mana” OR “Hylaeus mauiensis” OR “Hylaeus melanothrix” OR “Hylaeus nalo” OR “Hylaeus niloticus” OR “Hylaeus ombrias” OR “Hylaeus paradoxicus” OR “Hylaeus perspicuous” OR “Hylaeus psammobius” OR “Hylaeus satelles” OR “Hylaeus simplex” OR “Hylaeus solaris” OR “Macropis steironema opaca” OR “Osmia ashmeadii” OR “Osmia cascadica” OR “Perdita salicis euxantha” OR “Perdita salici sublaeta” OR “Perdita similes Pascoensis” OR “Perdita wyomingensis Sculleni” OR “Protandrena subdilatipes” OR “Sphecodogastra antiochensis”)

AND TITLE-ABS-KEY (“ecosystem servic*” OR “ecosystem function*” OR “pest regulat*” OR “disease regulat*” OR “pollin*” OR “nutrient cycl*” OR “soil form*” OR “climate regulat*” OR “natural-hazard regulat*” OR “water cycl*” OR “habitat provis*”)

AND TITLE-ABS-KEY (“farm*” OR “agri*” OR “food”)

AND TITLE-ABS-KEY (crop OR "harvest*" OR yield)

AND (EXCLUDE (PUBYEAR, 2022)) AND (LIMIT-TO (LANGUAGE, “English”))

AND NOT (aquatic OR marine OR livestock OR cattle OR pig OR bushmeat OR “wetland*” OR “peatland*” OR “medicin*”)

### Red list pollinating mammals (Regan et al., 2015)

#### General contribution

**- 4 documents**

TITLE-ABS-KEY (**“**Mystacina tuberculate” OR “Anoura cultrate” OR “Artibeus incomitatus” OR “Chiroderma improvisum” OR “Choeroniscus periosus” OR “Choeronycteris Mexicana” OR “Leptonycteris curasoae” OR “Leptonycteris nivalis” OR “Leptonycteris yerbabuenae” OR “Lonchophylla concave” OR “Lonchophylla dekeyseri” OR “Lonchophylla Hesperia” OR “Musonycteris harrisoni” OR “Platalina genovensium” OR “Platyrrhinus chocoensis” OR “Rhinophylla alethina” OR “Sturnira aratathomasi” OR “Sturnira mordax” OR “Sturnira nana” OR “Sturnira oporaphilum” OR “Sturnira thomasi” OR “Vampyressa Melissa” OR “Eidolon dupreanum” OR “Eidolon helvum” OR “Eonycteris robusta” OR “Epomophorus angolensis” OR “Myonycteris brachycephala” OR “Myonycteris relicta” OR “Notopteris macdonaldi” OR “Notopteris neocaledonica” OR “Pteropus aldabrensis” OR “Pteropus anetianus” OR “Pteropus aruensis” OR “Pteropus caniceps” OR “Pteropus capistratus” OR “Pteropus chrysoproctus” OR “Pteropus cognatus” OR “Pteropus dasymallus” OR “Pteropus faunulus” OR “Pteropus fundatus” OR “Pteropus livingstonii” OR “Pteropus lylei” OR “Pteropus mahaganus” OR “Pteropus mariannus” OR “Pteropus melanopogon” OR “Pteropus melanotus” OR “Pteropus molossinus” OR “Pteropus niger” OR “Pteropus nitendiensis” OR “Pteropus ocularis” OR “Pteropus ornatus” OR “Pteropus pelewensis” OR “Pteropus pohlei” OR “Pteropus poliocephalus” OR “Pteropus pselaphon” OR “Pteropus pumilus” OR “Pteropus rayneri” OR “Pteropus rennelli” OR “Pteropus rodricensis” OR “Pteropus rufus”

OR “Pteropus samoensis” OR “Pteropus temminckii” OR “Pteropus tuberculatus” OR “Pteropus ualanus” OR “Pteropus vampyrus” OR “Pteropus vetulus” OR “Pteropus voeltzkowi” OR “Pteropus woodfordi” OR “Pteropus yapensis” OR “Rousettus bidens” OR “Rousettus madagascariensis” OR “Rousettus obliviosus” OR “Rousettus spinalatus” OR “Syconycteris carolinae” OR “Syconycteris hobbit” OR “Dasyurus hallucatus” OR “Parantechinus apicalis” OR “Parantechinus apicalis” OR “Phascogale calura” OR “Phascogale tapoatafa”

OR “Burramys parvus” OR “Gymnobelideus leadbeateri” OR “Aotus lemurinus” OR “Ateles paniscus” OR “Brachyteles arachnoides” OR “Leontopithecus chrysopygus” OR “Callithrix flaviceps” OR “Saimiri oerstedii” OR “Cercopithecus diana” OR “Lophocebus aterrimus” OR “Macaca Silenus” OR “Mirza coquereli” OR “Daubentonia madagascariensis” OR “Eulemur fulvus” OR “Eulemur macaco” OR “Eulemur mongoz” OR “Eulemur rubriventer” OR “Varecia variegate” OR “Nycticebus coucang” OR “Plantacanthomys Lasiurus” OR “Sundasciurus hippurus”)

AND TITLE-ABS-KEY (“ecosystem servic*” OR “ecosystem funct*” OR “pest regulat*” OR “disease regulat*” OR “pollin*” OR “nutrient cycl*” OR “soil form*” OR “climate regulat*” OR “natural-hazard regulat*” OR “water cycl*” OR “habitat provis*”)

AND TITLE-ABS-KEY (“farm*” OR “agri*” OR “food”)

AND TITLE-ABS-KEY ("contribut*" OR "produc*" OR "return*" OR "profit*" OR revenue OR return OR output OR "incom*")

AND (EXCLUDE (PUBYEAR, 2022)) AND (LIMIT-TO (LANGUAGE, “English”))

AND NOT (aquatic OR marine OR livestock OR cattle OR pig OR bushmeat OR “wetland*” OR “peatland*” OR “medicin*”)

#### Unit measure contribution

**- 8 documents**

TITLE-ABS-KEY (**“**Mystacina tuberculate” OR “Anoura cultrate” OR “Artibeus incomitatus” OR “Chiroderma improvisum” OR “Choeroniscus periosus” OR “Choeronycteris Mexicana” OR “Leptonycteris curasoae” OR “Leptonycteris nivalis” OR “Leptonycteris yerbabuenae” OR “Lonchophylla concave” OR “Lonchophylla dekeyseri” OR “Lonchophylla Hesperia” OR “Musonycteris harrisoni” OR “Platalina genovensium” OR “Platyrrhinus chocoensis” OR “Rhinophylla alethina” OR “Sturnira aratathomasi” OR “Sturnira mordax” OR “Sturnira nana” OR “Sturnira oporaphilum” OR “Sturnira thomasi” OR “Vampyressa Melissa” OR “Eidolon dupreanum” OR “Eidolon helvum” OR “Eonycteris robusta” OR “Epomophorus angolensis” OR “Myonycteris brachycephala” OR “Myonycteris relicta” OR “Notopteris macdonaldi” OR “Notopteris neocaledonica” OR “Pteropus aldabrensis” OR “Pteropus anetianus” OR “Pteropus aruensis” OR “Pteropus caniceps” OR “Pteropus capistratus” OR “Pteropus chrysoproctus” OR “Pteropus cognatus” OR “Pteropus dasymallus” OR “Pteropus faunulus” OR “Pteropus fundatus” OR “Pteropus livingstonii” OR “Pteropus lylei” OR “Pteropus mahaganus” OR “Pteropus mariannus” OR “Pteropus melanopogon” OR “Pteropus melanotus” OR “Pteropus molossinus” OR “Pteropus niger” OR “Pteropus nitendiensis” OR “Pteropus ocularis” OR “Pteropus ornatus” OR “Pteropus pelewensis” OR “Pteropus pohlei” OR “Pteropus poliocephalus” OR “Pteropus pselaphon” OR “Pteropus pumilus” OR “Pteropus rayneri” OR “Pteropus rennelli” OR “Pteropus rodricensis” OR “Pteropus rufus”

OR “Pteropus samoensis” OR “Pteropus temminckii” OR “Pteropus tuberculatus” OR “Pteropus ualanus” OR “Pteropus vampyrus” OR “Pteropus vetulus” OR “Pteropus voeltzkowi” OR “Pteropus woodfordi” OR “Pteropus yapensis” OR “Rousettus bidens” OR “Rousettus madagascariensis” OR “Rousettus obliviosus” OR “Rousettus spinalatus” OR “Syconycteris carolinae” OR “Syconycteris hobbit” OR “Dasyurus hallucatus” OR “Parantechinus apicalis” OR “Parantechinus apicalis” OR “Phascogale calura” OR “Phascogale tapoatafa”

OR “Burramys parvus” OR “Gymnobelideus leadbeateri” OR “Aotus lemurinus” OR “Ateles paniscus” OR “Brachyteles arachnoides” OR “Leontopithecus chrysopygus” OR “Callithrix flaviceps” OR “Saimiri oerstedii” OR “Cercopithecus diana” OR “Lophocebus aterrimus” OR “Macaca Silenus” OR “Mirza coquereli” OR “Daubentonia madagascariensis” OR “Eulemur fulvus” OR “Eulemur macaco” OR “Eulemur mongoz” OR “Eulemur rubriventer” OR “Varecia variegate” OR “Nycticebus coucang” OR “Plantacanthomys Lasiurus” OR “Sundasciurus hippurus”)

AND TITLE-ABS-KEY (“ecosystem servic*” OR “ecosystem function*” OR “pest regulat*” OR “disease regulat*” OR “pollin*” OR “nutrient cycl*” OR “soil form*” OR “climate regulat*” OR “natural-hazard regulat*” OR “water cycl*” OR “habitat provis*”)

AND TITLE-ABS-KEY (“farm*” OR “agri*” OR “food”)

AND TITLE-ABS-KEY (kg OR tonnes OR "$" OR "dollar*" OR kcal OR calories OR hectare OR "ha" OR “ton*” OR “bushel*”)

AND (EXCLUDE (PUBYEAR, 2022)) AND (LIMIT-TO (LANGUAGE, “English”))

AND NOT (aquatic OR marine OR livestock OR cattle OR pig OR bushmeat OR “wetland*” OR “peatland*” OR “medicin*”)

#### Food contribution

**- 0 documents**

TITLE-ABS-KEY (**“**Mystacina tuberculate” OR “Anoura cultrate” OR “Artibeus incomitatus” OR “Chiroderma improvisum” OR “Choeroniscus periosus” OR “Choeronycteris Mexicana” OR “Leptonycteris curasoae” OR “Leptonycteris nivalis” OR “Leptonycteris yerbabuenae” OR “Lonchophylla concave” OR “Lonchophylla dekeyseri” OR “Lonchophylla Hesperia” OR “Musonycteris harrisoni” OR “Platalina genovensium” OR “Platyrrhinus chocoensis” OR “Rhinophylla alethina” OR “Sturnira aratathomasi” OR “Sturnira mordax” OR “Sturnira nana” OR “Sturnira oporaphilum” OR “Sturnira thomasi” OR “Vampyressa Melissa” OR “Eidolon dupreanum” OR “Eidolon helvum” OR “Eonycteris robusta” OR “Epomophorus angolensis” OR “Myonycteris brachycephala” OR “Myonycteris relicta” OR “Notopteris macdonaldi” OR “Notopteris neocaledonica” OR “Pteropus aldabrensis” OR “Pteropus anetianus” OR “Pteropus aruensis” OR “Pteropus caniceps” OR “Pteropus capistratus” OR “Pteropus chrysoproctus” OR “Pteropus cognatus” OR “Pteropus dasymallus” OR “Pteropus faunulus” OR “Pteropus fundatus” OR “Pteropus livingstonii” OR “Pteropus lylei” OR “Pteropus mahaganus” OR “Pteropus mariannus” OR “Pteropus melanopogon” OR “Pteropus melanotus” OR “Pteropus molossinus” OR “Pteropus niger” OR “Pteropus nitendiensis” OR “Pteropus ocularis” OR “Pteropus ornatus” OR “Pteropus pelewensis” OR “Pteropus pohlei” OR “Pteropus poliocephalus” OR “Pteropus pselaphon” OR “Pteropus pumilus” OR “Pteropus rayneri” OR “Pteropus rennelli” OR “Pteropus rodricensis” OR “Pteropus rufus”

OR “Pteropus samoensis” OR “Pteropus temminckii” OR “Pteropus tuberculatus” OR “Pteropus ualanus” OR “Pteropus vampyrus” OR “Pteropus vetulus” OR “Pteropus voeltzkowi” OR “Pteropus woodfordi” OR “Pteropus yapensis” OR “Rousettus bidens” OR “Rousettus madagascariensis” OR “Rousettus obliviosus” OR “Rousettus spinalatus” OR “Syconycteris carolinae” OR “Syconycteris hobbit” OR “Dasyurus hallucatus” OR “Parantechinus apicalis” OR “Parantechinus apicalis” OR “Phascogale calura” OR “Phascogale tapoatafa”

OR “Burramys parvus” OR “Gymnobelideus leadbeateri” OR “Aotus lemurinus” OR “Ateles paniscus” OR “Brachyteles arachnoides” OR “Leontopithecus chrysopygus” OR “Callithrix flaviceps” OR “Saimiri oerstedii” OR “Cercopithecus diana” OR “Lophocebus aterrimus” OR “Macaca Silenus” OR “Mirza coquereli” OR “Daubentonia madagascariensis” OR “Eulemur fulvus” OR “Eulemur macaco” OR “Eulemur mongoz” OR “Eulemur rubriventer” OR “Varecia variegate” OR “Nycticebus coucang” OR “Plantacanthomys Lasiurus” OR “Sundasciurus hippurus”)

AND TITLE-ABS-KEY (“ecosystem servic*” OR “ecosystem function*” OR “pest regulat*” OR “disease regulat*” OR “pollin*” OR “nutrient cycl*” OR “soil form*” OR “climate regulat*” OR “natural-hazard regulat*” OR “water cycl*” OR “habitat provis*”)

AND TITLE-ABS-KEY (“farm*” OR “agri*” OR “food”)

AND TITLE-ABS-KEY (crop OR "harvest*" OR yield)

AND (EXCLUDE (PUBYEAR, 2022)) AND (LIMIT-TO (LANGUAGE, “English”))

AND NOT (aquatic OR marine OR livestock OR cattle OR pig OR bushmeat OR “wetland*” OR “peatland*” OR “medicin*”)

### Red List pollinating birds (Regan et al., 2015)

#### General Contribution

**- 1 document**

TITLE-ABS-KEY (“Ramphodon naevius” OR “Glaucis dohrnii” OR “Phaethornis aethopygus” OR “Phaethornis koepckeae” OR “Augastes scutatus” OR” Augastes lumachella” OR “Heliangelus regalis” OR “Sephanoides fernandensis” OR “Discosura popelairii” OR “Lophornis gouldii” OR “Lophornis brachylophus” OR “Phlogophilus hemileucurus” OR “Phlogophilus harterti” OR” Aglaiocercus berlepschi” OR “Taphrolesbia griseiventris” OR “Oreotrochilus adela” OR “Ramphomicron dorsale” OR “Oxypogon cyanolaemus” OR “Oxypogon stuebelii” OR “Metallura iracunda” OR “Metallura baroni” OR “Haplophaedia lugens” OR “Eriocnemis nigrivestis” OR “Eriocnemis isabellae” OR “Eriocnemis derbyi” OR “Eriocnemis godini” OR “Eriocnemis cupreoventris” OR “Eriocnemis mirabilis” OR “Loddigesia mirabilis” OR “Aglaeactis aliciae” OR “Coeligena prunellei” OR “Coeligena orina” OR “Coeligena consita” OR “Heliodoxa gularis” OR “Cynanthus lawrencei” OR “Anthocephala floriceps” OR “Campylopterus ensipennis” OR “Campylopterus phainopeplus” OR “Campylopterus villaviscensio” OR “Eupherusa cyanophrys” OR “Eupherusa poliocerca” OR “Thalurania ridgwayi” OR ” Thalurania watertonii” OR “Amazilia castaneiventris” OR “Amazilia luciae” OR “Amazilia boucardi” OR “Goethalsia bella” OR “Lepidopyga lilliae” OR “Hylonympha macrocerca” OR “Eulidia yarrellii” OR “Chaetocercus bombus” OR “Chaetocercus berlepschi” OR “Doricha eliza” OR “Mellisuga helenae” OR “Selasphorus ardens” OR “Nestor meridionalis” OR “Brotogeris pyrrhoptera” OR “Pionites leucogaster” OR “Lathamus discolor” OR “Charmosyna palmarum” OR “Charmosyna meeki” OR “Charmosyna toxopei” OR “Charmosyna multistriata” OR “Charmosyna diadema” OR “Charmosyna amabilis” OR “Charmosyna margarethae” OR “Vini kuhlii” OR “Vini stepheni” OR “Vini peruviana” OR “Vini ultramarine” OR “Lorius garrulus” OR “Lorius domicella” OR “Lorius albidinucha” OR “Psitteuteles iris” OR “Eos histrio” OR “Eos reticulata” OR “Eos cyanogenia” OR “Trichoglossus forsteni” OR “Trichoglossus weberi” OR “Trichoglossus rosenbergii” OR “Trichoglossus johnstoniae” OR “Trichoglossus rubiginosus” OR “Lophornis gouldii” OR “Loriculus catamene” OR “Loriculus tener” OR “Loriculus exilis” OR “Loriculus pusillus” OR “Loriculus flosculus” OR “Elaenia ridleyana” OR “Notiomystis cincta” OR “Gymnomyza samoensis” OR “Gymnomyza aubryana” OR “Manorina melanotis” OR “Philemon brassi” OR “Philemon fuscicapillus” OR “Macgregoria pulchra” OR “Melidectes whitemanensis” OR “Melidectes princeps” OR “Lichmera notabilis” OR “Grantiella picta” OR “Myzomela kuehni” OR “Myzomela chermesina” OR “Myzomela malaitae” OR “Philesturnus carunculatus” OR “Vireo gracilirostris” OR “Zosterops mouroniensis” OR “Zosterops ficedulinus” OR “Zosterops griseovirescens” OR “Zosterops chloronothus” OR “Zosterops modestus” OR “Zosterops conspicillatus” OR “Zosterops rotensis” OR “Zosterops hypolais” OR “Zosterops natalis” OR “Zosterops flavus” OR “Zosterops grayi” OR “Zosterops uropygialis” OR “Zosterops somadikartai” OR “Zosterops nehrkorni” OR “Zosterops mysorensis” OR “Zosterops kuehni” OR “Zosterops vellalavella” OR “Zosterops splendidus” OR “Zosterops luteirostris” OR “Zosterops tenuirostris” OR “Zosterops albogularis” OR “Zosterops samoensis” OR “Zosterops oleaginous” OR “Woodfordia lacertosa” OR “Rukia longirostra” OR “Rukia ruki” OR “Cleptornis marchei” OR “Apalopteron familiar” OR “Madanga ruficollis” OR “Heleia muelleri” OR “Megazosterops palauensis” OR “Speirops melanocephalus” OR “Speirops brunneus” OR “Speirops leucophoeus” OR “Anthreptes reichenowi” OR “Anthreptes rhodolaemus” OR “Anthreptes pallidigaster” OR “Anthreptes rubritorques” OR “Nectarinia thomensis” OR “Nectarinia ursulae” OR “Nectarinia neergardi” OR “Nectarinia loveridgei” OR “Nectarinia moreaui” OR “Nectarinia rockefelleri” OR “Nectarinia rufipennis” OR “Aethopyga primigenia” OR “Aethopyga boltoni” OR “Aethopyga linaraborae” OR “Aethopyga duyvenbodei” OR “Hemignathus kauaiensis” OR “Hemignathus flavus” OR “Hemignathus parvus” OR “Hemignathus lucidus” OR “Hemignathus munroi” OR “Vestiaria coccinea” OR “Palmeria dolei” OR “Icterus oberi” OR “Icterus bonana” OR “Icterus laudabilis” OR “Thraupis cyanoptera” OR “Dacnis nigripes” OR “Conirostrum bicolor” OR “Diglossa venezuelensis” OR “Diglossa gloriosissima”)

AND TITLE-ABS-KEY (“ecosystem servic*” OR “ecosystem funct*” OR “pest regulat*” OR “disease regulat*” OR “pollin*” OR “nutrient cycl*” OR “soil form*” OR “climate regulat*” OR “natural-hazard regulat*” OR “water cycl*” OR “habitat provis*”)

AND TITLE-ABS-KEY (“farm*” OR “agri*” OR “food”)

AND TITLE-ABS-KEY ("contribut*" OR "produc*" OR "return*" OR "profit*" OR revenue OR return OR output OR "incom*")

AND (EXCLUDE (PUBYEAR, 2022)) AND (LIMIT-TO (LANGUAGE, “English”))

AND NOT (aquatic OR marine OR livestock OR cattle OR pig OR bushmeat OR “wetland*” OR “peatland*” OR “medicin*”)

#### Unit measure contribution

**- 1 document**

TITLE-ABS-KEY (“Ramphodon naevius” OR “Glaucis dohrnii” OR “Phaethornis aethopygus” OR “Phaethornis koepckeae” OR “Augastes scutatus” OR” Augastes lumachella” OR “Heliangelus regalis” OR “Sephanoides fernandensis” OR “Discosura popelairii” OR “Lophornis gouldii” OR “Lophornis brachylophus” OR “Phlogophilus hemileucurus” OR “Phlogophilus harterti” OR” Aglaiocercus berlepschi” OR “Taphrolesbia griseiventris” OR “Oreotrochilus adela” OR “Ramphomicron dorsale” OR “Oxypogon cyanolaemus” OR “Oxypogon stuebelii” OR “Metallura iracunda” OR “Metallura baroni” OR “Haplophaedia lugens” OR “Eriocnemis nigrivestis” OR “Eriocnemis isabellae” OR “Eriocnemis derbyi” OR “Eriocnemis godini” OR “Eriocnemis cupreoventris” OR “Eriocnemis mirabilis” OR “Loddigesia mirabilis” OR “Aglaeactis aliciae” OR “Coeligena prunellei” OR “Coeligena orina” OR “Coeligena consita” OR “Heliodoxa gularis” OR “Cynanthus lawrencei” OR “Anthocephala floriceps” OR “Campylopterus ensipennis” OR “Campylopterus phainopeplus” OR “Campylopterus villaviscensio” OR “Eupherusa cyanophrys” OR “Eupherusa poliocerca” OR “Thalurania ridgwayi” OR ” Thalurania watertonii” OR “Amazilia castaneiventris” OR “Amazilia luciae” OR “Amazilia boucardi” OR “Goethalsia bella” OR “Lepidopyga lilliae” OR “Hylonympha macrocerca” OR “Eulidia yarrellii” OR “Chaetocercus bombus” OR “Chaetocercus berlepschi” OR “Doricha eliza” OR “Mellisuga helenae” OR “Selasphorus ardens” OR “Nestor meridionalis” OR “Brotogeris pyrrhoptera” OR “Pionites leucogaster” OR “Lathamus discolor” OR “Charmosyna palmarum” OR “Charmosyna meeki” OR “Charmosyna toxopei” OR “Charmosyna multistriata” OR “Charmosyna diadema” OR “Charmosyna amabilis” OR “Charmosyna margarethae” OR “Vini kuhlii” OR “Vini stepheni” OR “Vini peruviana” OR “Vini ultramarine” OR “Lorius garrulus” OR “Lorius domicella” OR “Lorius albidinucha” OR “Psitteuteles iris” OR “Eos histrio” OR “Eos reticulata” OR “Eos cyanogenia” OR “Trichoglossus forsteni” OR “Trichoglossus weberi” OR “Trichoglossus rosenbergii” OR “Trichoglossus johnstoniae” OR “Trichoglossus rubiginosus” OR “Lophornis gouldii” OR “Loriculus catamene” OR “Loriculus tener” OR “Loriculus exilis” OR “Loriculus pusillus” OR “Loriculus flosculus” OR “Elaenia ridleyana” OR “Notiomystis cincta” OR “Gymnomyza samoensis” OR “Gymnomyza aubryana” OR “Manorina melanotis” OR “Philemon brassi” OR “Philemon fuscicapillus” OR “Macgregoria pulchra” OR “Melidectes whitemanensis” OR “Melidectes princeps” OR “Lichmera notabilis” OR “Grantiella picta” OR “Myzomela kuehni” OR “Myzomela chermesina” OR “Myzomela malaitae” OR “Philesturnus carunculatus” OR “Vireo gracilirostris” OR “Zosterops mouroniensis” OR “Zosterops ficedulinus” OR “Zosterops griseovirescens” OR “Zosterops chloronothus” OR “Zosterops modestus” OR “Zosterops conspicillatus” OR “Zosterops rotensis” OR “Zosterops hypolais” OR “Zosterops natalis” OR “Zosterops flavus” OR “Zosterops grayi” OR “Zosterops uropygialis” OR “Zosterops somadikartai” OR “Zosterops nehrkorni” OR “Zosterops mysorensis” OR “Zosterops kuehni” OR “Zosterops vellalavella” OR “Zosterops splendidus” OR “Zosterops luteirostris” OR “Zosterops tenuirostris” OR “Zosterops albogularis” OR “Zosterops samoensis” OR “Zosterops oleaginous” OR “Woodfordia lacertosa” OR “Rukia longirostra” OR “Rukia ruki” OR “Cleptornis marchei” OR “Apalopteron familiar” OR “Madanga ruficollis” OR “Heleia muelleri” OR “Megazosterops palauensis” OR “Speirops melanocephalus” OR “Speirops brunneus” OR “Speirops leucophoeus” OR “Anthreptes reichenowi” OR “Anthreptes rhodolaemus” OR “Anthreptes pallidigaster” OR “Anthreptes rubritorques” OR “Nectarinia thomensis” OR “Nectarinia ursulae” OR “Nectarinia neergardi” OR “Nectarinia loveridgei” OR “Nectarinia moreaui” OR “Nectarinia rockefelleri” OR “Nectarinia rufipennis” OR “Aethopyga primigenia” OR “Aethopyga boltoni” OR “Aethopyga linaraborae” OR “Aethopyga duyvenbodei” OR “Hemignathus kauaiensis” OR “Hemignathus flavus” OR “Hemignathus parvus” OR “Hemignathus lucidus” OR “Hemignathus munroi” OR “Vestiaria coccinea” OR “Palmeria dolei” OR “Icterus oberi” OR “Icterus bonana” OR “Icterus laudabilis” OR “Thraupis cyanoptera” OR “Dacnis nigripes” OR “Conirostrum bicolor” OR “Diglossa venezuelensis” OR “Diglossa gloriosissima”)

AND TITLE-ABS-KEY (“ecosystem servic*” OR “ecosystem function*” OR “pest regulat*” OR “disease regulat*” OR “pollin*” OR “nutrient cycl*” OR “soil form*” OR “climate regulat*” OR “natural-hazard regulat*” OR “water cycl*” OR “habitat provis*”)

AND TITLE-ABS-KEY (“farm*” OR “agri*” OR “food”)

AND TITLE-ABS-KEY (crop OR "harvest*" OR yield)

AND (EXCLUDE (PUBYEAR, 2022)) AND (LIMIT-TO (LANGUAGE, “English”))

AND NOT (aquatic OR marine OR livestock OR cattle OR pig OR bushmeat OR “wetland*” OR “peatland*” OR “medicin*”)

#### Food contribution

**- 1 document**

TITLE-ABS-KEY (“Ramphodon naevius” OR “Glaucis dohrnii” OR “Phaethornis aethopygus” OR “Phaethornis koepckeae” OR “Augastes scutatus” OR” Augastes lumachella” OR “Heliangelus regalis” OR “Sephanoides fernandensis” OR “Discosura popelairii” OR “Lophornis gouldii” OR “Lophornis brachylophus” OR “Phlogophilus hemileucurus” OR “Phlogophilus harterti” OR” Aglaiocercus berlepschi” OR “Taphrolesbia griseiventris” OR “Oreotrochilus adela” OR “Ramphomicron dorsale” OR “Oxypogon cyanolaemus” OR “Oxypogon stuebelii” OR “Metallura iracunda” OR “Metallura baroni” OR “Haplophaedia lugens” OR “Eriocnemis nigrivestis” OR “Eriocnemis isabellae” OR “Eriocnemis derbyi” OR “Eriocnemis godini” OR “Eriocnemis cupreoventris” OR “Eriocnemis mirabilis” OR “Loddigesia mirabilis” OR “Aglaeactis aliciae” OR “Coeligena prunellei” OR “Coeligena orina” OR “Coeligena consita” OR “Heliodoxa gularis” OR “Cynanthus lawrencei” OR “Anthocephala floriceps” OR “Campylopterus ensipennis” OR “Campylopterus phainopeplus” OR “Campylopterus villaviscensio” OR “Eupherusa cyanophrys” OR “Eupherusa poliocerca” OR “Thalurania ridgwayi” OR ” Thalurania watertonii” OR “Amazilia castaneiventris” OR “Amazilia luciae” OR “Amazilia boucardi” OR “Goethalsia bella” OR “Lepidopyga lilliae” OR “Hylonympha macrocerca” OR “Eulidia yarrellii” OR “Chaetocercus bombus” OR “Chaetocercus berlepschi” OR “Doricha eliza” OR “Mellisuga helenae” OR “Selasphorus ardens” OR “Nestor meridionalis” OR “Brotogeris pyrrhoptera” OR “Pionites leucogaster” OR “Lathamus discolor” OR “Charmosyna palmarum” OR “Charmosyna meeki” OR “Charmosyna toxopei” OR “Charmosyna multistriata” OR “Charmosyna diadema” OR “Charmosyna amabilis” OR “Charmosyna margarethae” OR “Vini kuhlii” OR “Vini stepheni” OR “Vini peruviana” OR “Vini ultramarine” OR “Lorius garrulus” OR “Lorius domicella” OR “Lorius albidinucha” OR “Psitteuteles iris” OR “Eos histrio” OR “Eos reticulata” OR “Eos cyanogenia” OR “Trichoglossus forsteni” OR “Trichoglossus weberi” OR “Trichoglossus rosenbergii” OR “Trichoglossus johnstoniae” OR “Trichoglossus rubiginosus” OR “Lophornis gouldii” OR “Loriculus catamene” OR “Loriculus tener” OR “Loriculus exilis” OR “Loriculus pusillus” OR “Loriculus flosculus” OR “Elaenia ridleyana” OR “Notiomystis cincta” OR “Gymnomyza samoensis” OR “Gymnomyza aubryana” OR “Manorina melanotis” OR “Philemon brassi” OR “Philemon fuscicapillus” OR “Macgregoria pulchra” OR “Melidectes whitemanensis” OR “Melidectes princeps” OR “Lichmera notabilis” OR “Grantiella picta” OR “Myzomela kuehni” OR “Myzomela chermesina” OR “Myzomela malaitae” OR “Philesturnus carunculatus” OR “Vireo gracilirostris” OR “Zosterops mouroniensis” OR “Zosterops ficedulinus” OR “Zosterops griseovirescens” OR “Zosterops chloronothus” OR “Zosterops modestus” OR “Zosterops conspicillatus” OR “Zosterops rotensis” OR “Zosterops hypolais” OR “Zosterops natalis” OR “Zosterops flavus” OR “Zosterops grayi” OR “Zosterops uropygialis” OR “Zosterops somadikartai” OR “Zosterops nehrkorni” OR “Zosterops mysorensis” OR “Zosterops kuehni” OR “Zosterops vellalavella” OR “Zosterops splendidus” OR “Zosterops luteirostris” OR “Zosterops tenuirostris” OR “Zosterops albogularis” OR “Zosterops samoensis” OR “Zosterops oleaginous” OR “Woodfordia lacertosa” OR “Rukia longirostra” OR “Rukia ruki” OR “Cleptornis marchei” OR “Apalopteron familiar” OR “Madanga ruficollis” OR “Heleia muelleri” OR “Megazosterops palauensis” OR “Speirops melanocephalus” OR “Speirops brunneus” OR “Speirops leucophoeus” OR “Anthreptes reichenowi” OR “Anthreptes rhodolaemus” OR “Anthreptes pallidigaster” OR “Anthreptes rubritorques” OR “Nectarinia thomensis” OR “Nectarinia ursulae” OR “Nectarinia neergardi” OR “Nectarinia loveridgei” OR “Nectarinia moreaui” OR “Nectarinia rockefelleri” OR “Nectarinia rufipennis” OR “Aethopyga primigenia” OR “Aethopyga boltoni” OR “Aethopyga linaraborae” OR “Aethopyga duyvenbodei” OR “Hemignathus kauaiensis” OR “Hemignathus flavus” OR “Hemignathus parvus” OR “Hemignathus lucidus” OR “Hemignathus munroi” OR “Vestiaria coccinea” OR “Palmeria dolei” OR “Icterus oberi” OR “Icterus bonana” OR “Icterus laudabilis” OR “Thraupis cyanoptera” OR “Dacnis nigripes” OR “Conirostrum bicolor” OR “Diglossa venezuelensis” OR “Diglossa gloriosissima”)

AND TITLE-ABS-KEY (“ecosystem servic*” OR “ecosystem function*” OR “pest regulat*” OR “disease regulat*” OR “pollin*” OR “nutrient cycl*” OR “soil form*” OR “climate regulat*” OR “natural-hazard regulat*” OR “water cycl*” OR “habitat provis*”)

AND TITLE-ABS-KEY (“farm*” OR “agri*” OR “food”)

AND TITLE-ABS-KEY (crop OR "harvest*" OR yield)

AND (EXCLUDE (PUBYEAR, 2022)) AND (LIMIT-TO (LANGUAGE, “English”))

AND NOT (aquatic OR marine OR livestock OR cattle OR pig OR bushmeat OR “wetland*” OR “peatland*” OR “medicin*”)

## Web of science - English

### IUCN

#### General contribution

**- 421 documents**

AB= (IUCN OR rare OR "endanger*" OR vulnerable OR "threaten*")

AND AB= (“ecosystem servic*” OR “ecosystem function*”)

AND AB= (“farm*” OR “agri*” OR “food”)

AND AB= ("contribut*" OR "produc*" OR "return*" OR "profit*" OR revenue OR return OR output OR "incom*")

NOT AB=(aquatic OR marine OR livestock OR cattle OR pig OR bushmeat OR “wetland*” OR “peatland*” OR “medicin*”)

**- 298 documents**

AB= (IUCN OR rare OR "endanger*" OR vulnerable OR "threaten*")

AND AB= (“pest regulat*” OR “disease regulat*” OR “pollin*” OR “nutrient cycl*” OR “soil form*” OR “climate regulat*” OR “natural-hazard regulat*” OR “water cycl*” OR “habitat provis*”)

AND AB= (“farm*” OR “agri*” OR “food”)

AND AB= ("contribut*" OR "produc*" OR "return*" OR "profit*" OR revenue OR return OR output OR "incom*")

NOT AB=(aquatic OR marine OR livestock OR cattle OR pig OR bushmeat OR “wetland*” OR “peatland*” OR “medicin*”)

**- 3 documents**

TI= (IUCN OR rare OR "endanger*" OR vulnerable OR "threaten*")

AND TI= (“ecosystem servic*” OR “ecosystem function*” OR “pest regulat*” OR “disease regulat*” OR “pollin*” OR “nutrient cycl*” OR “soil form*” OR “climate regulat*” OR “natural-hazard regulat*” OR “water cycl*” OR “habitat provis*”)

AND TI= (“farm*” OR “agri*” OR “food”)

AND TI= ("contribut*" OR "produc*" OR "return*" OR "profit*" OR revenue OR return OR output OR "incom*")

NOT TI=(aquatic OR marine OR livestock OR cattle OR pig OR bushmeat OR “wetland*” OR “peatland*” OR “medicin*”)

#### Unit measure contribution

**- 86 documents**

AB= (IUCN OR rare OR "endanger*" OR vulnerable OR "threaten*")

AND AB= (“ecosystem servic*” OR “ecosystem function*” OR “pest regulat*” OR “disease regulat*” OR “pollin*” OR “nutrient cycl*” OR “soil form*” OR “climate regulat*” OR “natural-hazard regulat*” OR “water cycl*” OR “habitat provis*”)

AND AB= (“farm*” OR “agri*” OR “food”)

AND AB= (kg OR tonnes OR "dollar*" OR kcal OR calories OR hectare OR "ha" OR “ton*” OR “bushel*”)

NOT AB=(aquatic OR marine OR livestock OR cattle OR pig OR bushmeat OR “wetland*” OR “peatland*” OR “medicin*”)

**- 0 documents**

TI= (IUCN OR rare OR "endanger*" OR vulnerable OR "threaten*")

AND TI=(“ecosystem servic*” OR “ecosystem function*” OR “pest regulat*” OR “disease regulat*” OR “pollin*” OR “nutrient cycl*” OR “soil form*” OR “climate regulat*” OR “natural-hazard regulat*” OR “water cycl*” OR “habitat provis*”)

AND TI= (“farm*” OR “agri*” OR “food”)

AND TI= (kg OR tonnes OR "dollar*" OR kcal OR calories OR hectare OR "ha" OR “ton*” OR “bushel*”)

NOT TI=(aquatic OR marine OR livestock OR cattle OR pig OR bushmeat OR “wetland*” OR “peatland*” OR “medicin*”)

#### Food contribution

**- 383 documents**

AB= (IUCN OR rare OR "endanger*" OR vulnerable OR "threaten*")

AND AB= (“ecosystem servic*” OR “ecosystem function*” OR “pest regulat*” OR “disease regulat*” OR “pollin*” OR “nutrient cycl*” OR “soil form*” OR “climate regulat*” OR “natural-hazard regulat*” OR “water cycl*” OR “habitat provis*”)

AND AB= (“farm*” OR “agri*” OR “food”)

AND AB= (crop OR "harvest*" OR yield)

NOT AB=(aquatic OR marine OR livestock OR cattle OR pig OR bushmeat OR “wetland*” OR “peatland*” OR “medicin*”)

**- 2 documents**

TI= (IUCN OR rare OR "endanger*" OR vulnerable OR "threaten*")

AND TI= (“ecosystem servic*” OR “ecosystem function*” OR “pest regulat*” OR “disease regulat*” OR “pollin*” OR “nutrient cycl*” OR “soil form*” OR “climate regulat*” OR “natural-hazard regulat*” OR “water cycl*” OR “habitat provis*”)

AND TI= (“farm*” OR “agri*” OR “food”)

AND TI= (crop OR "harvest*" OR yield)

NOT TI=(aquatic OR marine OR livestock OR cattle OR pig OR bushmeat OR “wetland*” OR “peatland*” OR “medicin*”)

### AREA

#### General contribution

**- 402 documents**

**AB= ("small distribution" OR "small range" OR "small extent" OR "small niche" OR "small habitat" OR "small area" OR "limit*distribution" OR "limit* range" OR "limit* extent" OR "limit* niche" OR "limit* habitat" OR "limit* area*" OR "restrict* distribution" OR "restrict* range" OR "restrict* extent" OR "restrict* niche" OR "restrict* habitat" OR "restrict* area*" OR "confin* distribution" OR "confin* range" OR "confin* extent" OR "confin* niche" OR "confin* habitat" OR "confin* area*" OR "declin* distribution" OR "declin* range" OR "declin* extent" OR "declin* habitat" OR "declin* niche" OR "declin* habitat" OR "declin* area" OR "decreas* distribution" OR "decreas* range" OR "decreas* extent" OR "decreas* habitat" OR "decreas* niche" OR "decreas* habitat" OR "decreas* area" OR ("loss " AND "distribution") OR ("loss " AND "range ") OR ("loss " AND "extent") OR ("loss " AND "niche") OR ("loss" AND "habitat"))**

AND AB= (“ecosystem servic*” OR “ecosystem function*” OR “pest regulat*” OR “disease regulat*” OR “pollin*” OR “nutrient cycl*” OR “soil form*” OR “climate regulat*” OR “natural-hazard regulat*” OR “water cycl*” OR “habitat provis*”)

AND AB= (“farm*” OR “agri*” OR “food”)

AND AB=("contribut*" OR "produc*" OR "return*" OR "profit*" OR revenue OR return OR output OR "incom*")

NOT AB=(aquatic OR marine OR livestock OR cattle OR pig OR bushmeat OR “wetland*” OR “peatland*” OR “medicin*”)

**- 0 documents**

**TI= ("small distribution" OR "small range" OR "small extent" OR "small niche" OR "small habitat" OR "small area" OR "limit*distribution" OR "limit* range" OR "limit* extent" OR "limit* niche" OR "limit* habitat" OR "limit* area*" OR "restrict*distribution" OR "restrict* range" OR "restrict* extent" OR "restrict* niche" OR "restrict* habitat" OR "restrict* area*" OR "confin* distribution" OR "confin* range" OR "confin* extent" OR "confin* niche" OR "confin* habitat" OR "confin* area*" OR "declin* distribution" OR "declin* range" OR "declin* extent" OR "declin* habitat" OR "declin* niche" OR "declin* habitat" OR "declin* area" OR "decreas* distribution" OR "decreas* range" OR "decreas* extent" OR "decreas* habitat" OR "decreas* niche" OR "decreas* habitat" OR "decreas* area" OR ("loss " AND "distribution") OR ("loss " AND "range ") OR ("loss " AND "extent") OR ("loss " AND "niche") OR ("loss" AND "habitat"))**

AND TI= (“ecosystem servic*” OR “ecosystem function*” OR “pest regulat*” OR “disease regulat*” OR “pollin*” OR “nutrient cycl*” OR “soil form*” OR “climate regulat*” OR “natural-hazard regulat*” OR “water cycl*” OR “habitat provis*”)

AND TI= (“farm*” OR “agri*” OR “food”)

AND TI=("contribut*" OR "produc*" OR "return*" OR "profit*" OR revenue OR return OR output OR "incom*")

NOT TI=(aquatic OR marine OR livestock OR cattle OR pig OR bushmeat OR “wetland*” OR “peatland*” OR “medicin*”)

**- 203 documents**

AB= (**"loss " AND "area")**

AND AB=(“ecosystem servic*” OR “ecosystem function*” OR “pest regulat*” OR “disease regulat*” OR “pollin*” OR “nutrient cycl*” OR “soil form*” OR “climate regulat*” OR “natural-hazard regulat*” OR “water cycl*” OR “habitat provis*”)

AND AB= (“farm*” OR “agri*” OR “food”)

AND AB=("contribut*" OR "produc*" OR "return*" OR "profit*" OR revenue OR return OR output OR "incom*")

NOT AB=(aquatic OR marine OR livestock OR cattle OR pig OR bushmeat OR “wetland*” OR “peatland*” OR “medicin*”)

**- 1 document**

TI= (**"loss " AND "area")**

AND TI=(“ecosystem servic*” OR “ecosystem function*” OR “pest regulat*” OR “disease regulat*” OR “pollin*” OR “nutrient cycl*” OR “soil form*” OR “climate regulat*” OR “natural-hazard regulat*” OR “water cycl*” OR “habitat provis*”)

AND TI= (“farm*” OR “agri*” OR “food”)

AND TI=("contribut*" OR "produc*" OR "return*" OR "profit*" OR revenue OR return OR output OR "incom*")

NOT TI=(aquatic OR marine OR livestock OR cattle OR pig OR bushmeat OR “wetland*” OR “peatland*” OR “medicin*”)

#### Unit measure contribution

**- 117 documents**

AB= ("small distribution" OR "small range" OR "small extent" OR "small niche" OR "small habitat" OR "small area" OR "limit*distribution" OR "limit* range" OR "limit* extent" OR "limit* niche" OR "limit* habitat" OR "limit* area*" OR "restrict*distribution" OR "restrict* range" OR "restrict* extent" OR "restrict* niche" OR "restrict* habitat" OR "restrict* area*" OR "confin* distribution" OR "confin* range" OR "confin* extent" OR "confin* niche" OR "confin* habitat" OR "confin* area*" OR "declin* distribution" OR "declin* range" OR "declin* extent" OR "declin* habitat" OR "declin* niche" OR "declin* habitat" OR "declin* area" OR "decreas* distribution" OR "decreas* range" OR "decreas* extent" OR "decreas* habitat" OR "decreas* niche" OR "decreas* habitat" OR "decreas* area" OR ("loss " AND "distribution") OR ("loss " AND "range ") OR ("loss " AND "extent") OR ("loss " AND "niche") OR ("loss" AND "habitat") OR ("loss " AND "area"))

AND AB= (“ecosystem servic*” OR “ecosystem function*” OR “pest regulat*” OR “disease regulat*” OR “pollin*” OR “nutrient cycl*” OR “soil form*” OR “climate regulat*” OR “natural-hazard regulat*” OR “water cycl*” OR “habitat provis*”)

AND AB= (“farm*” OR “agri*” OR “food”)

AND AB= (kg OR tonnes OR "dollar*" OR kcal OR calories OR hectare OR "ha" OR “ton*” OR “bushel*”)

NOT AB=(aquatic OR marine OR livestock OR cattle OR pig OR bushmeat OR “wetland*” OR “peatland*” OR “medicin*”)

**- 0 documents**

TI= ("small distribution" OR "small range" OR "small extent" OR "small niche" OR "small habitat" OR "small area" OR "limit*distribution" OR "limit* range" OR "limit* extent" OR "limit* niche" OR "limit* habitat" OR "limit* area*" OR "restrict*distribution" OR "restrict* range" OR "restrict* extent" OR "restrict* niche" OR "restrict* habitat" OR "restrict* area*" OR "confin* distribution" OR "confin* range" OR "confin* extent" OR "confin* niche" OR "confin* habitat" OR "confin* area*" OR "declin* distribution" OR "declin* range" OR "declin* extent" OR "declin* habitat" OR "declin* niche" OR "declin* habitat" OR "declin* area" OR "decreas* distribution" OR "decreas* range" OR "decreas* extent" OR "decreas* habitat" OR "decreas* niche" OR "decreas* habitat" OR "decreas* area" OR ("loss " AND "distribution") OR ("loss " AND "range ") OR ("loss " AND "extent") OR ("loss " AND "niche") OR ("loss" AND "habitat") OR ("loss " AND "area"))

AND TI= (“ecosystem servic*” OR “ecosystem function*” OR “pest regulat*” OR “disease regulat*” OR “pollin*” OR “nutrient cycl*” OR “soil form*” OR “climate regulat*” OR “natural-hazard regulat*” OR “water cycl*” OR “habitat provis*”)

AND TI= (“farm*” OR “agri*” OR “food”)

AND TI= (kg OR tonnes OR "dollar*" OR kcal OR calories OR hectare OR "ha" OR “ton*” OR “bushel*”)

NOT TI=(aquatic OR marine OR livestock OR cattle OR pig OR bushmeat OR “wetland*” OR “peatland*” OR “medicin*”)

#### Food contribution

**- 301 documents**

AB= ("small distribution" OR "small range" OR "small extent" OR "small niche" OR "small habitat" OR "small area" OR "limit*distribution" OR "limit* range" OR "limit* extent" OR "limit* niche" OR "limit* habitat" OR "limit* area*" OR "restrict*distribution" OR "restrict* range" OR "restrict* extent" OR "restrict* niche" OR "restrict* habitat" OR "restrict* area*" OR "confin* distribution" OR "confin* range" OR "confin* extent" OR "confin* niche" OR "confin* habitat" OR "confin* area*" OR "declin* distribution" OR "declin* range" OR "declin* extent" OR "declin* habitat" OR "declin* niche" OR "declin* habitat" OR "declin* area" OR "decreas* distribution" OR "decreas* range" OR "decreas* extent" OR "decreas* habitat" OR "decreas* niche" OR "decreas* habitat" OR "decreas* area" OR ("loss " AND "distribution") OR ("loss " AND "range ") OR ("loss " AND "extent") OR ("loss " AND "niche") OR ("loss" AND "habitat") OR ("loss " AND "area"))

AND AB= (“ecosystem servic*” OR “ecosystem function*” OR “pest regulat*” OR “disease regulat*” OR “pollin*” OR “nutrient cycl*” OR “soil form*” OR “climate regulat*” OR “natural-hazard regulat*” OR “water cycl*” OR “habitat provis*”)

AND AB= (“farm*” OR “agri*” OR “food”)

AND AB= (crop OR "harvest*" OR yield)

NOT AB=(aquatic OR marine OR livestock OR cattle OR pig OR bushmeat OR “wetland*” OR “peatland*” OR “medicin*”)

**- 0 documents**

TI= ("small distribution" OR "small range" OR "small extent" OR "small niche" OR "small habitat" OR "small area" OR "limit*distribution" OR "limit* range" OR "limit* extent" OR "limit* niche" OR "limit* habitat" OR "limit* area*" OR "restrict*distribution" OR "restrict* range" OR "restrict* extent" OR "restrict* niche" OR "restrict* habitat" OR "restrict* area*" OR "confin* distribution" OR "confin* range" OR "confin* extent" OR "confin* niche" OR "confin* habitat" OR "confin* area*" OR "declin* distribution" OR "declin* range" OR "declin* extent" OR "declin* habitat" OR "declin* niche" OR "declin* habitat" OR "declin* area" OR "decreas* distribution" OR "decreas* range" OR "decreas* extent" OR "decreas* habitat" OR "decreas* niche" OR "decreas* habitat" OR "decreas* area" OR ("loss " AND "distribution") OR ("loss " AND "range ") OR ("loss " AND "extent") OR ("loss " AND "niche") OR ("loss" AND "habitat") OR ("loss " AND "area"))

AND TI= (“ecosystem servic*” OR “ecosystem function*” OR “pest regulat*” OR “disease regulat*” OR “pollin*” OR “nutrient cycl*” OR “soil form*” OR “climate regulat*” OR “natural-hazard regulat*” OR “water cycl*” OR “habitat provis*”)

AND TI= (“farm*” OR “agri*” OR “food”)

AND TI= (crop OR "harvest*" OR yield)

NOT TI=(aquatic OR marine OR livestock OR cattle OR pig OR bushmeat OR “wetland*” OR “peatland*” OR “medicin*”)

### ABUNDANCE

#### General contribution

**- 325 documents**

**AB= ("low abundance" OR "low number*" OR "low population" OR “low density” OR "small abundance" OR "small numbers" OR "small population" OR "few individuals" OR "few populations" OR "limit* abundance" OR "limit* number*" OR "limit* population*" OR "restrict* abundance" OR "restrict* number*" OR "restrict*population*" OR "confin* abundance" OR "confin* number*" OR "confin* population*" OR "declin* abundance" OR "declin* number*" OR "declin* population*" OR "decreas* abundance" OR "decreas* number*" OR "decreas* population*" OR "fall* abundance" OR "fall* number*" OR ("loss " AND "abundance") OR ("loss " AND "number*") OR ("loss " AND "individual*"))**

AND AB=(“ecosystem servic*” OR “ecosystem function*” OR “pest regulat*” OR “disease regulat*” OR “pollin*” OR “nutrient cycl*” OR “soil form*” OR “climate regulat*” OR “natural-hazard regulat*” OR “water cycl*” OR “habitat provis*”)

AND AB= (“farm*” OR “agri*” OR “food”)

AND AB= ("contribut*" OR "produc*" OR "return*" OR "profit*" OR revenue OR return OR output OR "incom*")

NOT AB=(aquatic OR marine OR livestock OR cattle OR pig OR bushmeat OR “wetland*” OR “peatland*” OR “medicin*”)

**- 0 documents**

**TI= ("low abundance" OR "low number*" OR "low population" OR “low density” OR "small abundance" OR "small numbers" OR "small population" OR "few individuals" OR "few populations" OR "limit* abundance" OR "limit* number*" OR "limit* population*" OR "restrict* abundance" OR "restrict* number*" OR "restrict*population*" OR "confin* abundance" OR "confin* number*" OR "confin* population*" OR "declin* abundance" OR "declin* number*" OR "declin* population*" OR "decreas* abundance" OR "decreas* number*" OR "decreas* population*" OR "fall* abundance" OR "fall* number*" OR ("loss " AND "abundance") OR ("loss " AND "number*") OR ("loss " AND "individual*"))**

AND TI=(“ecosystem servic*” OR “ecosystem function*” OR “pest regulat*” OR “disease regulat*” OR “pollin*” OR “nutrient cycl*” OR “soil form*” OR “climate regulat*” OR “natural-hazard regulat*” OR “water cycl*” OR “habitat provis*”)

AND TI= (“farm*” OR “agri*” OR “food”)

AND TI= ("contribut*" OR "produc*" OR "return*" OR "profit*" OR revenue OR return OR output OR "incom*")

NOT TI=(aquatic OR marine OR livestock OR cattle OR pig OR bushmeat OR “wetland*” OR “peatland*” OR “medicin*”)

- **208 documents**

AB= **("loss " AND "population*")**

AND AB= (“ecosystem servic*” OR “ecosystem function*” OR “pest regulat*” OR “disease regulat*” OR “pollin*” OR “nutrient cycl*” OR “soil form*” OR “climate regulat*” OR “natural-hazard regulat*” OR “water cycl*” OR “habitat provis*”)

AND AB= (“farm*” OR “agri*” OR “food”)

AND AB= ("contribut*" OR "produc*" OR "return*" OR "profit*" OR revenue OR return OR output OR "incom*")

NOT AB=(aquatic OR marine OR livestock OR cattle OR pig OR bushmeat OR “wetland*” OR “peatland*” OR “medicin*”)

-  **0 documents**

TI= **("loss " AND "population*")**

AND TI= (“ecosystem servic*” OR “ecosystem function*” OR “pest regulat*” OR “disease regulat*” OR “pollin*” OR “nutrient cycl*” OR “soil form*” OR “climate regulat*” OR “natural-hazard regulat*” OR “water cycl*” OR “habitat provis*”)

AND TI= (“farm*” OR “agri*” OR “food”)

AND TI= ("contribut*" OR "produc*" OR "return*" OR "profit*" OR revenue OR return OR output OR "incom*")

NOT TI=(aquatic OR marine OR livestock OR cattle OR pig OR bushmeat OR “wetland*” OR “peatland*” OR “medicin*”)

#### Unit measure contribution

**- 89 documents**

AB= ("low abundance" OR "low number*" OR "low population" OR “low density” OR "small abundance" OR "small numbers" OR "small population" OR "few individuals" OR "few populations" OR "limit* abundance" OR "limit* number*" OR "limit*population*" OR "restrict* abundance" OR "restrict* number*" OR "restrict* population*" OR "confin* abundance" OR "confin* number*" OR "confin* population*" OR "declin* abundance" OR "declin* number*" OR "declin* population*" OR "decreas* abundance" OR "decreas* number*" OR "decreas* population*" OR "fall* abundance" OR "fall* number*" OR ("loss " AND "abundance") OR ("loss " AND "number*") OR ("loss " AND "individual*") OR ("loss " AND "population*"))

AND AB=(“ecosystem servic*” OR “ecosystem function*” OR “pest regulat*” OR “disease regulat*” OR “pollin*” OR “nutrient cycl*” OR “soil form*” OR “climate regulat*” OR “natural-hazard regulat*” OR “water cycl*” OR “habitat provis*”)

AND AB= (“farm*” OR “agri*” OR “food”)

AND AB= (kg OR tonnes OR "dollar*" OR kcal OR calories OR hectare OR "ha" OR “ton*” OR “bushel*”)

NOT AB=(aquatic OR marine OR livestock OR cattle OR pig OR bushmeat OR “wetland*” OR “peatland*” OR “medicin*”)

**- 0 documents**

TI= ("low abundance" OR "low number*" OR "low population" OR “low density” OR "small abundance" OR "small numbers" OR "small population" OR "few individuals" OR "few populations" OR "limit* abundance" OR "limit* number*" OR "limit*population*" OR "restrict* abundance" OR "restrict* number*" OR "restrict* population*" OR "confin* abundance" OR "confin* number*" OR "confin* population*" OR "declin* abundance" OR "declin* number*" OR "declin* population*" OR "decreas* abundance" OR "decreas* number*" OR "decreas* population*" OR "fall* abundance" OR "fall* number*" OR ("loss " AND "abundance") OR ("loss " AND "number*") OR ("loss " AND "individual*") OR ("loss " AND "population*"))

AND TI=(“ecosystem servic*” OR “ecosystem function*” OR “pest regulat*” OR “disease regulat*” OR “pollin*” OR “nutrient cycl*” OR “soil form*” OR “climate regulat*” OR “natural-hazard regulat*” OR “water cycl*” OR “habitat provis*”)

AND TI= (“farm*” OR “agri*” OR “food”)

AND TI= (kg OR tonnes OR "dollar*" OR kcal OR calories OR hectare OR "ha" OR “ton*” OR “bushel*”)

NOT TI=(aquatic OR marine OR livestock OR cattle OR pig OR bushmeat OR “wetland*” OR “peatland*” OR “medicin*”)

#### Food contribution

**- 313 documents**

AB= ("low abundance" OR "low number*" OR "low population" OR “low density” OR "small abundance" OR "small numbers" OR "small population" OR "few individuals" OR "few populations" OR "limit* abundance" OR "limit* number*" OR "limit*population*" OR "restrict* abundance" OR "restrict* number*" OR "restrict* population*" OR "confin* abundance" OR "confin* number*" OR "confin* population*" OR "declin* abundance" OR "declin* number*" OR "declin* population*" OR "decreas* abundance" OR "decreas* number*" OR "decreas* population*" OR "fall* abundance" OR "fall* number*" OR ("loss " AND "abundance") OR ("loss " AND "number*") OR ("loss " AND "individual*") OR ("loss " AND "population*"))

AND AB=(“ecosystem servic*” OR “ecosystem function*” OR “pest regulat*” OR “disease regulat*” OR “pollin*” OR “nutrient cycl*” OR “soil form*” OR “climate regulat*” OR “natural-hazard regulat*” OR “water cycl*” OR “habitat provis*”)

AND AB= (“farm*” OR “agri*” OR “food”)

AND AB= (crop OR "harvest*" OR yield)

NOT AB=(aquatic OR marine OR livestock OR cattle OR pig OR bushmeat OR “wetland*” OR “peatland*” OR “medicin*”)

**- 0 documents**

TI= ("low abundance" OR "low number*" OR "low population" OR “low density” OR "small abundance" OR "small numbers" OR "small population" OR "few individuals" OR "few populations" OR "limit* abundance" OR "limit* number*" OR "limit*population*" OR "restrict* abundance" OR "restrict* number*" OR "restrict* population*" OR "confin* abundance" OR "confin* number*" OR "confin* population*" OR "declin* abundance" OR "declin* number*" OR "declin* population*" OR "decreas* abundance" OR "decreas* number*" OR "decreas* population*" OR "fall* abundance" OR "fall* number*" OR ("loss " AND "abundance") OR ("loss " AND "number*") OR ("loss " AND "individual*") OR ("loss " AND "population*"))

AND TI=(“ecosystem servic*” OR “ecosystem function*” OR “pest regulat*” OR “disease regulat*” OR “pollin*” OR “nutrient cycl*” OR “soil form*” OR “climate regulat*” OR “natural-hazard regulat*” OR “water cycl*” OR “habitat provis*”)

AND TI= (“farm*” OR “agri*” OR “food”)

AND TI= (crop OR "harvest*" OR yield)

NOT TI=(aquatic OR marine OR livestock OR cattle OR pig OR bushmeat OR “wetland*” OR “peatland*” OR “medicin*”)

### European Red List of bees (Nieto et al., 2014)

**- 2 documents**

AB=(“Ammobates dusmeti” OR “Andrena labiatula” OR “Andrena ornata” OR “Andrena tridentata” OR “Bombus cullumanus” OR “Megachile cypricola” OR “Nomada siciliensis” OR “Ammobates electoides” OR “Ammobatoides abdominalis” OR “Andrena comta” OR “Colletes wolfi” OR “Dasypoda braccata” OR “Andrena magna” OR “Andrena stepposa” OR “Andrena stigmatica ” OR “Bombus armeniacus” OR “Bombus brodmannicus” OR “Bombus fragrans” OR “Bombus inexspectatus” OR “Bombus mocsaryi” OR “Bombus reinigiellus” OR “Bombus zonatus” OR “Colletes anchusae” OR “Colletes caspicus” OR “Colletes collaris” OR “Colletes graeffei” OR “Colletes merceti” OR “Colletes meyeri” OR “Colletes punctatus” OR “Colletes sierrensis” OR “Dasypoda frieseana” OR “Dasypoda spinigera” OR “Dasypoda suripes” OR “Flavipanurgus granadensis” OR “Halictus arinthiacus” OR “Halictus microcardia” OR “Halictus semitectus” OR “Icteranthidium cimbiciforme” OR “Lasioglossum breviventre” OR “Lasioglossum laeve” OR “Lasioglossum quadrisig natum” OR “Lasioglossum sexmaculatum” OR “Lasioglossum sexnotatulum” OR “Lasioglossum soror” OR “Lasioglossum subfasciatum” OR “Lasioglossum virens” OR “Melitta melanura” OR “Nomada italica” OR “Nomada pulchra” OR “Osmia maritima” OR “Parammobatodes minutus” OR “Trachusa interrupta” OR “Andrena transitoria” OR “Biastes truncatus” OR “Bombus alpinus” OR “Bombus confusus” OR “Bombus distinguendus” OR “Bombus gerstaeckeri” OR “Bombus hyperboreus” OR “Bombus muscorum”

OR “Bombus polaris” OR “Bombus pomorum” OR “Coelioxys elongatula” OR “Colletes chengtehensis” OR “Colletes dimidiatus” OR “Colletes floralis” OR “Colletes fodiens” OR “Colletes impunctatus” OR “Colletes moricei” OR “Colletes perezi” OR “Colletes pulchellus” OR “Halictus leucaheneus” OR “Melitta hispanica” OR “Melitta kastiliensis” OR “Nomada noskiewiczi” OR “Systropha planidens” OR “Andrena nanaeformis”)

AND AB= (“ecosystem servic*” OR “ecosystem funct*” OR “pest regulat*” OR “disease regulat*” OR “pollin*” OR “nutrient cycl*” OR “soil form*” OR “climate regulat*” OR “natural-hazard regulat*” OR “water cycl*” OR “habitat provis*”)

AND AB= (“farm*” OR “agri*” OR “food”)

AND AB= ("contribut*" OR "produc*" OR "return*" OR "profit*" OR revenue OR return OR output OR "incom*" OR kg OR tonnes OR "dollar*" OR kcal OR calories OR hectare OR "ha" OR “ton*” OR “bushel*” OR crop OR "harvest*" OR yield)

NOT AB=(aquatic OR marine OR livestock OR cattle OR pig OR bushmeat OR “wetland*” OR “peatland*” OR “medicin*”)

**- 0 documents**

TI=(“Ammobates dusmeti” OR “Andrena labiatula” OR “Andrena ornata” OR “Andrena tridentata” OR “Bombus cullumanus” OR “Megachile cypricola” OR “Nomada siciliensis” OR “Ammobates electoides” OR “Ammobatoides abdominalis” OR “Andrena comta” OR “Colletes wolfi” OR “Dasypoda braccata” OR “Andrena magna” OR “Andrena stepposa” OR “Andrena stigmatica ” OR “Bombus armeniacus” OR “Bombus brodmannicus” OR “Bombus fragrans” OR “Bombus inexspectatus” OR “Bombus mocsaryi” OR “Bombus reinigiellus” OR “Bombus zonatus” OR “Colletes anchusae” OR “Colletes caspicus” OR “Colletes collaris” OR “Colletes graeffei” OR “Colletes merceti” OR “Colletes meyeri” OR “Colletes punctatus” OR “Colletes sierrensis” OR “Dasypoda frieseana” OR “Dasypoda spinigera” OR “Dasypoda suripes” OR “Flavipanurgus granadensis” OR “Halictus arinthiacus” OR “Halictus microcardia” OR “Halictus semitectus” OR “Icteranthidium cimbiciforme” OR “Lasioglossum breviventre” OR “Lasioglossum laeve” OR “Lasioglossum quadrisig natum” OR “Lasioglossum sexmaculatum” OR “Lasioglossum sexnotatulum” OR “Lasioglossum soror” OR “Lasioglossum subfasciatum” OR “Lasioglossum virens” OR “Melitta melanura” OR “Nomada italica” OR “Nomada pulchra” OR “Osmia maritima” OR “Parammobatodes minutus” OR “Trachusa interrupta” OR “Andrena transitoria” OR “Biastes truncatus” OR “Bombus alpinus” OR “Bombus confusus” OR “Bombus distinguendus” OR “Bombus gerstaeckeri” OR “Bombus hyperboreus” OR “Bombus muscorum”

OR “Bombus polaris” OR “Bombus pomorum” OR “Coelioxys elongatula” OR “Colletes chengtehensis” OR “Colletes dimidiatus” OR “Colletes floralis” OR “Colletes fodiens” OR “Colletes impunctatus” OR “Colletes moricei” OR “Colletes perezi” OR “Colletes pulchellus” OR “Halictus leucaheneus” OR “Melitta hispanica” OR “Melitta kastiliensis” OR “Nomada noskiewiczi” OR “Systropha planidens” OR “Andrena nanaeformis”)

AND TI= (“ecosystem servic*” OR “ecosystem funct*” OR “pest regulat*” OR “disease regulat*” OR “pollin*” OR “nutrient cycl*” OR “soil form*” OR “climate regulat*” OR “natural-hazard regulat*” OR “water cycl*” OR “habitat provis*”)

AND TI= (“farm*” OR “agri*” OR “food”)

AND TI= ("contribut*" OR "produc*" OR "return*" OR "profit*" OR revenue OR return OR output OR "incom*" OR kg OR tonnes OR "dollar*" OR kcal OR calories OR hectare OR "ha" OR “ton*” OR “bushel*” OR crop OR "harvest*" OR yield)

NOT TI=(aquatic OR marine OR livestock OR cattle OR pig OR bushmeat OR “wetland*” OR “peatland*” OR “medicin*”)

### Red listed pollinating insects of North America (National Research Council, 2007)

**- 6 documents**

AB=(“Agathymus evansi” OR “Amblyscirtes linda” OR “Apodemia mormo langei” OR “Atrytone arogos” OR “Boloria acrocnema” OR “Boloria alberta” OR “Calephelis borealis” OR “Callophrys comstocki” OR “Callophrys irus” OR “Callophrys lanoraieensis” OR “Callophrys mossii bayensis” OR “Celotes limpia” OR “Cyclargus thomasi bethunebakeri” OR “Erora laeta” OR “Erynnis persius persius” OR “Euchloe ausonides insulana” OR “Euphilotes battoides allyni” OR “Euphilotes baueri” OR “Euphilotes enoptes smithi” OR “Euphilotes mojave” OR “Euphydryas anicia cloudcrofti” OR “Euphydryas editha bayensis” OR “Euphydryas editha quino” OR “Euphydryas editha taylori” OR “Euphydryas gillettii” OR “Euphyes bayensis” OR “Euphyes dukesi” OR “Euproserpinus Euterpe” OR “Fixsenia [Satyrium] polingi” OR “Glaucopsyche lygdamus palosverdesensis” OR “Heraclides aristodemus ponceanus” OR “Hesperia dacotae” OR “Hesperia leonardus montana” OR “Hesperia ottoe” OR “Hesperopsis gracielae” OR “Icaricia icarioides fender” OR “Icaricia icarioides missionensis” OR “Lycaeides idas lotis” OR “Lycaeides melissa samuelis” OR “Manduca blackburni” OR “Mitoura hesseli” OR “Neonympha mitchellii francisci” OR “Neonympha mitchellii mitchellii” OR “Oarisma powesheik” OR “Papilio joanae” OR “Polites mardon” OR “Problema bulenta” OR “Problema byssus” OR “Pseudocopaeodes eunus obscurus” OR “Pyrgus ruralis lagunae” OR “Satyrium kingi” OR “Speyeria callippe callippe” OR “Speyeria diana” OR “Speyeria idalia” OR “Speyeria zerene behrensii” OR “Speyeria zerene Hippolyta” OR “Speyeria zerene myrtleae” OR “Stallingsia maculosus” OR “Andrena aculeata” OR “Andrena winnemuccana” OR “Ashmeadiella sculleni” OR “Bombus affinis” OR “Bombus franklini” OR “Bombus lucorum” OR “Bombus occidentalis” OR “Bombus terricola” OR “Calliopsis barri” OR “Epeoloides pilosula” OR “Eucera douglasiana” OR “Eucera frater lata” OR “Halictus harmonius“ OR “Halictus pinguismentus” OR “Hesperapis kayella” OR “Hoplitis orthognathus” OR “Hoplitis producta subgracilis” OR “Hylaeus akoko” OR “Hylaeus anomalus” OR “Hylaeus anthracinus” OR “Hylaeus assimulans” OR “Hylaeus dimidiatus” OR “Hylaeus facilis” OR “Hylaeus finitimus” OR “Hylaeus flavifrons” OR “Hylaeus gliddenae” OR “Hylaeus hilaris” OR “Hylaeus hula” OR “Hylaeus kona” OR “Hylaeus kuakea” OR “Hylaeus longiceps” OR “Hylaeus lunicraterius” OR “Hylaeus mana” OR “Hylaeus mauiensis” OR “Hylaeus melanothrix” OR “Hylaeus nalo” OR “Hylaeus niloticus” OR “Hylaeus ombrias” OR “Hylaeus paradoxicus” OR “Hylaeus perspicuous” OR “Hylaeus psammobius” OR “Hylaeus satelles”

OR “Hylaeus simplex” OR “Hylaeus solaris” OR “Macropis steironema opaca” OR “Osmia ashmeadii” OR “Osmia cascadica” OR “Perdita salicis euxantha” OR “Perdita salici sublaeta” OR “Perdita similes Pascoensis” OR “Perdita wyomingensis Sculleni” OR “Protandrena subdilatipes” OR “Sphecodogastra antiochensis”)

AND AB= (“ecosystem servic*” OR “ecosystem function*” OR “pest regulat*” OR “disease regulat*” OR “pollin*” OR “nutrient cycl*” OR “soil form*” OR “climate regulat*” OR “natural-hazard regulat*” OR “water cycl*” OR “habitat provis*”)

AND AB= (“farm*” OR “agri*” OR “food”)

AND AB= ("contribut*" OR "produc*" OR "return*" OR "profit*" OR revenue OR return OR output OR "incom*" OR kg OR tonnes OR "dollar*" OR kcal OR calories OR hectare OR "ha" OR “ton*” OR “bushel*” OR crop OR "harvest*" OR yield)

NOT AB=(aquatic OR marine OR livestock OR cattle OR pig OR bushmeat OR “wetland*” OR “peatland*” OR “medicin*”)

- **0 documents**

TI=(“Agathymus evansi” OR “Amblyscirtes linda” OR “Apodemia mormo langei” OR “Atrytone arogos” OR “Boloria acrocnema” OR “Boloria alberta” OR “Calephelis borealis” OR “Callophrys comstocki” OR “Callophrys irus” OR “Callophrys lanoraieensis” OR “Callophrys mossii bayensis” OR “Celotes limpia” OR “Cyclargus thomasi bethunebakeri” OR “Erora laeta” OR “Erynnis persius persius” OR “Euchloe ausonides insulana” OR “Euphilotes battoides allyni” OR “Euphilotes baueri” OR “Euphilotes enoptes smithi” OR “Euphilotes mojave” OR “Euphydryas anicia cloudcrofti” OR “Euphydryas editha bayensis” OR “Euphydryas editha quino” OR “Euphydryas editha taylori” OR “Euphydryas gillettii” OR “Euphyes bayensis” OR “Euphyes dukesi” OR “Euproserpinus Euterpe” OR “Fixsenia [Satyrium] polingi” OR “Glaucopsyche lygdamus palosverdesensis” OR “Heraclides aristodemus ponceanus” OR “Hesperia dacotae” OR “Hesperia leonardus montana” OR “Hesperia ottoe” OR “Hesperopsis gracielae” OR “Icaricia icarioides fender” OR “Icaricia icarioides missionensis” OR “Lycaeides idas lotis” OR “Lycaeides melissa samuelis” OR “Manduca blackburni” OR “Mitoura hesseli” OR “Neonympha mitchellii francisci” OR “Neonympha mitchellii mitchellii” OR “Oarisma powesheik” OR “Papilio joanae” OR “Polites mardon” OR “Problema bulenta” OR “Problema byssus” OR “Pseudocopaeodes eunus obscurus” OR “Pyrgus ruralis lagunae” OR “Satyrium kingi” OR “Speyeria callippe callippe” OR “Speyeria diana” OR “Speyeria idalia” OR “Speyeria zerene behrensii” OR “Speyeria zerene Hippolyta” OR “Speyeria zerene myrtleae” OR “Stallingsia maculosus” OR “Andrena aculeata” OR “Andrena winnemuccana” OR “Ashmeadiella sculleni” OR “Bombus affinis” OR “Bombus franklini” OR “Bombus lucorum” OR “Bombus occidentalis” OR “Bombus terricola” OR “Calliopsis barri” OR “Epeoloides pilosula” OR “Eucera douglasiana” OR “Eucera frater lata” OR “Halictus harmonius“ OR “Halictus pinguismentus” OR “Hesperapis kayella” OR “Hoplitis orthognathus” OR “Hoplitis producta subgracilis” OR “Hylaeus akoko” OR “Hylaeus anomalus” OR “Hylaeus anthracinus” OR “Hylaeus assimulans” OR “Hylaeus dimidiatus” OR “Hylaeus facilis” OR “Hylaeus finitimus” OR “Hylaeus flavifrons” OR “Hylaeus gliddenae” OR “Hylaeus hilaris” OR “Hylaeus hula” OR “Hylaeus kona” OR “Hylaeus kuakea” OR “Hylaeus longiceps” OR “Hylaeus lunicraterius” OR “Hylaeus mana” OR “Hylaeus mauiensis” OR “Hylaeus melanothrix” OR “Hylaeus nalo” OR “Hylaeus niloticus” OR “Hylaeus ombrias” OR “Hylaeus paradoxicus” OR “Hylaeus perspicuous” OR “Hylaeus psammobius” OR “Hylaeus satelles”

OR “Hylaeus simplex” OR “Hylaeus solaris” OR “Macropis steironema opaca” OR “Osmia ashmeadii” OR “Osmia cascadica” OR “Perdita salicis euxantha” OR “Perdita salici sublaeta” OR “Perdita similes Pascoensis” OR “Perdita wyomingensis Sculleni” OR “Protandrena subdilatipes” OR “Sphecodogastra antiochensis”)

AND TI= (“ecosystem servic*” OR “ecosystem function*” OR “pest regulat*” OR “disease regulat*” OR “pollin*” OR “nutrient cycl*” OR “soil form*” OR “climate regulat*” OR “natural-hazard regulat*” OR “water cycl*” OR “habitat provis*”)

AND TI= (“farm*” OR “agri*” OR “food”)

AND TI= ("contribut*" OR "produc*" OR "return*" OR "profit*" OR revenue OR return OR output OR "incom*" OR kg OR tonnes OR "dollar*" OR kcal OR calories OR hectare OR "ha" OR “ton*” OR “bushel*” OR crop OR "harvest*" OR yield)

NOT TI=(aquatic OR marine OR livestock OR cattle OR pig OR bushmeat OR “wetland*” OR “peatland*” OR “medicin*”)

### Red list pollinating mammals (Regan et al., 2015)

**- 6 documents**

AB=(**“**Mystacina tuberculate” OR “Anoura cultrate” OR “Artibeus incomitatus” OR “Chiroderma improvisum” OR “Choeroniscus periosus” OR “Choeronycteris Mexicana” OR “Leptonycteris curasoae” OR “Leptonycteris nivalis” OR “Leptonycteris yerbabuenae” OR “Lonchophylla concave” OR “Lonchophylla dekeyseri” OR “Lonchophylla Hesperia” OR “Musonycteris harrisoni” OR “Platalina genovensium” OR “Platyrrhinus chocoensis” OR “Rhinophylla alethina” OR “Sturnira aratathomasi” OR “Sturnira mordax” OR “Sturnira nana” OR “Sturnira oporaphilum” OR “Sturnira thomasi” OR “Vampyressa Melissa” OR “Eidolon dupreanum” OR “Eidolon helvum” OR “Eonycteris robusta” OR “Epomophorus angolensis” OR “Myonycteris brachycephala” OR “Myonycteris relicta” OR “Notopteris macdonaldi” OR “Notopteris neocaledonica” OR “Pteropus aldabrensis” OR “Pteropus anetianus” OR “Pteropus aruensis” OR “Pteropus caniceps” OR “Pteropus capistratus” OR “Pteropus chrysoproctus” OR “Pteropus cognatus” OR “Pteropus dasymallus” OR “Pteropus faunulus” OR “Pteropus fundatus” OR “Pteropus livingstonii” OR “Pteropus lylei” OR “Pteropus mahaganus” OR “Pteropus mariannus” OR “Pteropus melanopogon” OR “Pteropus melanotus” OR “Pteropus molossinus” OR “Pteropus niger” OR “Pteropus nitendiensis” OR “Pteropus ocularis” OR “Pteropus ornatus” OR “Pteropus pelewensis” OR “Pteropus pohlei” OR “Pteropus poliocephalus” OR “Pteropus pselaphon” OR “Pteropus pumilus” OR “Pteropus rayneri” OR “Pteropus rennelli” OR “Pteropus rodricensis” OR “Pteropus rufus” OR “Pteropus samoensis” OR “Pteropus temminckii” OR “Pteropus tuberculatus” OR “Pteropus ualanus” OR “Pteropus vampyrus” OR “Pteropus vetulus” OR “Pteropus voeltzkowi” OR “Pteropus woodfordi” OR “Pteropus yapensis” OR “Rousettus bidens” OR “Rousettus madagascariensis” OR “Rousettus obliviosus” OR “Rousettus spinalatus” OR “Syconycteris carolinae” OR “Syconycteris hobbit” OR “Dasyurus hallucatus” OR “Parantechinus apicalis” OR “Parantechinus apicalis” OR “Phascogale calura” OR “Phascogale tapoatafa” OR “Burramys parvus” OR “Gymnobelideus leadbeateri” OR “Aotus lemurinus” OR “Ateles paniscus” OR “Brachyteles arachnoides” OR “Leontopithecus chrysopygus” OR “Callithrix flaviceps” OR “Saimiri oerstedii” OR “Cercopithecus diana” OR “Lophocebus aterrimus” OR “Macaca Silenus” OR “Mirza coquereli” OR “Daubentonia madagascariensis” OR “Eulemur fulvus” OR “Eulemur macaco” OR “Eulemur mongoz” OR “Eulemur rubriventer” OR “Varecia variegate” OR “Nycticebus coucang” OR “Plantacanthomys Lasiurus” OR “Sundasciurus hippurus”)

AND AB= (“ecosystem servic*” OR “ecosystem function*” OR “pest regulat*” OR “disease regulat*” OR “pollin*” OR “nutrient cycl*” OR “soil form*” OR “climate regulat*” OR “natural-hazard regulat*” OR “water cycl*” OR “habitat provis*”)

AND AB= (“farm*” OR “agri*” OR “food”)

AND AB= ("contribut*" OR "produc*" OR "return*" OR "profit*" OR revenue OR return OR output OR "incom*" OR kg OR tonnes OR "dollar*" OR kcal OR calories OR hectare OR "ha" OR “ton*” OR “bushel*” OR crop OR "harvest*" OR yield)

NOT AB=(aquatic OR marine OR livestock OR cattle OR pig OR bushmeat OR “wetland*” OR “peatland*” OR “medicin*”)

**- 0 documents**

TI=(**“**Mystacina tuberculate” OR “Anoura cultrate” OR “Artibeus incomitatus” OR “Chiroderma improvisum” OR “Choeroniscus periosus” OR “Choeronycteris Mexicana” OR “Leptonycteris curasoae” OR “Leptonycteris nivalis” OR “Leptonycteris yerbabuenae” OR “Lonchophylla concave” OR “Lonchophylla dekeyseri” OR “Lonchophylla Hesperia” OR “Musonycteris harrisoni” OR “Platalina genovensium” OR “Platyrrhinus chocoensis” OR “Rhinophylla alethina” OR “Sturnira aratathomasi” OR “Sturnira mordax” OR “Sturnira nana” OR “Sturnira oporaphilum” OR “Sturnira thomasi” OR “Vampyressa Melissa” OR “Eidolon dupreanum” OR “Eidolon helvum” OR “Eonycteris robusta” OR “Epomophorus angolensis” OR “Myonycteris brachycephala” OR “Myonycteris relicta” OR “Notopteris macdonaldi” OR “Notopteris neocaledonica” OR “Pteropus aldabrensis” OR “Pteropus anetianus” OR “Pteropus aruensis” OR “Pteropus caniceps” OR “Pteropus capistratus” OR “Pteropus chrysoproctus” OR “Pteropus cognatus” OR “Pteropus dasymallus” OR “Pteropus faunulus” OR “Pteropus fundatus” OR “Pteropus livingstonii” OR “Pteropus lylei” OR “Pteropus mahaganus” OR “Pteropus mariannus” OR “Pteropus melanopogon” OR “Pteropus melanotus” OR “Pteropus molossinus” OR “Pteropus niger” OR “Pteropus nitendiensis” OR “Pteropus ocularis” OR “Pteropus ornatus” OR “Pteropus pelewensis” OR “Pteropus pohlei” OR “Pteropus poliocephalus” OR “Pteropus pselaphon” OR “Pteropus pumilus” OR “Pteropus rayneri” OR “Pteropus rennelli” OR “Pteropus rodricensis” OR “Pteropus rufus” OR “Pteropus samoensis” OR “Pteropus temminckii” OR “Pteropus tuberculatus” OR “Pteropus ualanus” OR “Pteropus vampyrus” OR “Pteropus vetulus” OR “Pteropus voeltzkowi” OR “Pteropus woodfordi” OR “Pteropus yapensis” OR “Rousettus bidens” OR “Rousettus madagascariensis” OR “Rousettus obliviosus” OR “Rousettus spinalatus” OR “Syconycteris carolinae” OR “Syconycteris hobbit” OR “Dasyurus hallucatus” OR “Parantechinus apicalis” OR “Parantechinus apicalis” OR “Phascogale calura” OR “Phascogale tapoatafa” OR “Burramys parvus” OR “Gymnobelideus leadbeateri” OR “Aotus lemurinus” OR “Ateles paniscus” OR “Brachyteles arachnoides” OR “Leontopithecus chrysopygus” OR “Callithrix flaviceps” OR “Saimiri oerstedii” OR “Cercopithecus diana” OR “Lophocebus aterrimus” OR “Macaca Silenus” OR “Mirza coquereli” OR “Daubentonia madagascariensis” OR “Eulemur fulvus” OR “Eulemur macaco” OR “Eulemur mongoz” OR “Eulemur rubriventer” OR “Varecia variegate” OR “Nycticebus coucang” OR “Plantacanthomys Lasiurus” OR “Sundasciurus hippurus”)

AND TI= (“ecosystem servic*” OR “ecosystem function*” OR “pest regulat*” OR “disease regulat*” OR “pollin*” OR “nutrient cycl*” OR “soil form*” OR “climate regulat*” OR “natural-hazard regulat*” OR “water cycl*” OR “habitat provis*”)

AND TI= (“farm*” OR “agri*” OR “food”)

AND TI= ("contribut*" OR "produc*" OR "return*" OR "profit*" OR revenue OR return OR output OR "incom*" OR kg OR tonnes OR "dollar*" OR kcal OR calories OR hectare OR "ha" OR “ton*” OR “bushel*” OR crop OR "harvest*" OR yield)

NOT TI=(aquatic OR marine OR livestock OR cattle OR pig OR bushmeat OR “wetland*” OR “peatland*” OR “medicin*”)

### Red List pollinating birds (Regan et al., 2015)

**- 2 documents**

AB=(“Ramphodon naevius” OR “Glaucis dohrnii” OR “Phaethornis aethopygus” OR “Phaethornis koepckeae” OR “Augastes scutatus” OR” Augastes lumachella” OR “Heliangelus regalis” OR “Sephanoides fernandensis” OR “Discosura popelairii” OR “Lophornis gouldii” OR “Lophornis brachylophus” OR “Phlogophilus hemileucurus” OR “Phlogophilus harterti” OR” Aglaiocercus berlepschi” OR “Taphrolesbia griseiventris” OR “Oreotrochilus adela” OR “Ramphomicron dorsale” OR “Oxypogon cyanolaemus” OR “Oxypogon stuebelii” OR “Metallura iracunda” OR “Metallura baroni” OR “Haplophaedia lugens” OR “Eriocnemis nigrivestis” OR “Eriocnemis isabellae” OR “Eriocnemis derbyi” OR “Eriocnemis godini” OR “Eriocnemis cupreoventris” OR “Eriocnemis mirabilis” OR “Loddigesia mirabilis” OR “Aglaeactis aliciae” OR “Coeligena prunellei” OR “Coeligena orina” OR “Coeligena consita” OR “Heliodoxa gularis” OR “Cynanthus lawrencei” OR “Anthocephala floriceps” OR “Campylopterus ensipennis” OR “Campylopterus phainopeplus” OR “Campylopterus villaviscensio” OR “Eupherusa cyanophrys” OR “Eupherusa poliocerca” OR “Thalurania ridgwayi” OR ” Thalurania watertonii” OR “Amazilia castaneiventris” OR “Amazilia luciae” OR “Amazilia boucardi” OR “Goethalsia bella” OR “Lepidopyga lilliae” OR “Hylonympha macrocerca” OR “Eulidia yarrellii” OR “Chaetocercus bombus” OR “Chaetocercus berlepschi” OR “Doricha eliza” OR “Mellisuga helenae” OR “Selasphorus ardens” OR “Nestor meridionalis” OR “Brotogeris pyrrhoptera” OR “Pionites leucogaster” OR “Lathamus discolor” OR “Charmosyna palmarum” OR “Charmosyna meeki” OR “Charmosyna toxopei” OR “Charmosyna multistriata” OR “Charmosyna diadema” OR “Charmosyna amabilis” OR “Charmosyna margarethae” OR “Vini kuhlii” OR “Vini stepheni” OR “Vini peruviana” OR “Vini ultramarine” OR “Lorius garrulus” OR “Lorius domicella” OR “Lorius albidinucha” OR “Psitteuteles iris” OR “Eos histrio” OR “Eos reticulata” OR “Eos cyanogenia” OR “Trichoglossus forsteni” OR “Trichoglossus weberi” OR “Trichoglossus rosenbergii” OR “Trichoglossus johnstoniae” OR “Trichoglossus rubiginosus” OR “Lophornis gouldii” OR “Loriculus catamene” OR “Loriculus tener” OR “Loriculus exilis” OR “Loriculus pusillus” OR “Loriculus flosculus” OR “Elaenia ridleyana” OR “Notiomystis cincta” OR “Gymnomyza samoensis” OR “Gymnomyza aubryana” OR “Manorina melanotis” OR “Philemon brassi” OR “Philemon fuscicapillus” OR “Macgregoria pulchra” OR “Melidectes whitemanensis” OR “Melidectes princeps” OR “Lichmera notabilis” OR “Grantiella picta” OR “Myzomela kuehni” OR “Myzomela chermesina” OR “Myzomela malaitae” OR “Philesturnus carunculatus” OR “Vireo gracilirostris” OR “Zosterops mouroniensis” OR “Zosterops ficedulinus” OR “Zosterops griseovirescens” OR “Zosterops chloronothus” OR “Zosterops modestus” OR “Zosterops conspicillatus” OR “Zosterops rotensis” OR “Zosterops hypolais” OR “Zosterops natalis” OR “Zosterops flavus” OR “Zosterops grayi” OR “Zosterops uropygialis” OR “Zosterops somadikartai” OR “Zosterops nehrkorni” OR “Zosterops mysorensis” OR “Zosterops kuehni” OR “Zosterops vellalavella” OR “Zosterops splendidus” OR “Zosterops luteirostris” OR “Zosterops tenuirostris” OR “Zosterops albogularis” OR “Zosterops samoensis” OR “Zosterops oleaginous” OR “Woodfordia lacertosa” OR “Rukia longirostra” OR “Rukia ruki” OR “Cleptornis marchei” OR “Apalopteron familiar” OR “Madanga ruficollis” OR “Heleia muelleri” OR “Megazosterops palauensis” OR “Speirops melanocephalus” OR “Speirops brunneus” OR “Speirops leucophoeus” OR “Anthreptes reichenowi” OR “Anthreptes rhodolaemus” OR “Anthreptes pallidigaster” OR “Anthreptes rubritorques” OR “Nectarinia thomensis” OR “Nectarinia ursulae” OR “Nectarinia neergardi” OR “Nectarinia loveridgei” OR “Nectarinia moreaui” OR “Nectarinia rockefelleri” OR “Nectarinia rufipennis” OR “Aethopyga primigenia” OR “Aethopyga boltoni” OR “Aethopyga linaraborae” OR “Aethopyga duyvenbodei” OR “Hemignathus kauaiensis” OR “Hemignathus flavus” OR “Hemignathus parvus” OR “Hemignathus lucidus” OR “Hemignathus munroi” OR “Vestiaria coccinea” OR “Palmeria dolei” OR “Icterus oberi” OR “Icterus bonana” OR “Icterus laudabilis” OR “Thraupis cyanoptera” OR “Dacnis nigripes” OR “Conirostrum bicolor” OR “Diglossa venezuelensis” OR “Diglossa gloriosissima”)

AND AB= (“ecosystem servic*” OR “ecosystem function*” OR “pest regulat*” OR “disease regulat*” OR “pollin*” OR “nutrient cycl*” OR “soil form*” OR “climate regulat*” OR “natural-hazard regulat*” OR “water cycl*” OR “habitat provis*”)

AND AB= (“farm*” OR “agri*” OR “food”)

AND AB= ("contribut*" OR "produc*" OR "return*" OR "profit*" OR revenue OR return OR output OR "incom*" OR kg OR tonnes OR "dollar*" OR kcal OR calories OR hectare OR "ha" OR “ton*” OR “bushel*” OR crop OR "harvest*" OR yield)

NOT AB=(aquatic OR marine OR livestock OR cattle OR pig OR bushmeat OR “wetland*” OR “peatland*” OR “medicin*”)

- **0 documents**

TI=(“Ramphodon naevius” OR “Glaucis dohrnii” OR “Phaethornis aethopygus” OR “Phaethornis koepckeae” OR “Augastes scutatus” OR” Augastes lumachella” OR “Heliangelus regalis” OR “Sephanoides fernandensis” OR “Discosura popelairii” OR “Lophornis gouldii” OR “Lophornis brachylophus” OR “Phlogophilus hemileucurus” OR “Phlogophilus harterti” OR” Aglaiocercus berlepschi” OR “Taphrolesbia griseiventris” OR “Oreotrochilus adela” OR “Ramphomicron dorsale” OR “Oxypogon cyanolaemus” OR “Oxypogon stuebelii” OR “Metallura iracunda” OR “Metallura baroni” OR “Haplophaedia lugens” OR “Eriocnemis nigrivestis” OR “Eriocnemis isabellae” OR “Eriocnemis derbyi” OR “Eriocnemis godini” OR “Eriocnemis cupreoventris” OR “Eriocnemis mirabilis” OR “Loddigesia mirabilis” OR “Aglaeactis aliciae” OR “Coeligena prunellei” OR “Coeligena orina” OR “Coeligena consita” OR “Heliodoxa gularis” OR “Cynanthus lawrencei” OR “Anthocephala floriceps” OR “Campylopterus ensipennis” OR “Campylopterus phainopeplus” OR “Campylopterus villaviscensio” OR “Eupherusa cyanophrys” OR “Eupherusa poliocerca” OR “Thalurania ridgwayi” OR ” Thalurania watertonii” OR “Amazilia castaneiventris” OR “Amazilia luciae” OR “Amazilia boucardi” OR “Goethalsia bella” OR “Lepidopyga lilliae” OR “Hylonympha macrocerca” OR “Eulidia yarrellii” OR “Chaetocercus bombus” OR “Chaetocercus berlepschi” OR “Doricha eliza” OR “Mellisuga helenae” OR “Selasphorus ardens” OR “Nestor meridionalis” OR “Brotogeris pyrrhoptera” OR “Pionites leucogaster” OR “Lathamus discolor” OR “Charmosyna palmarum” OR “Charmosyna meeki” OR “Charmosyna toxopei” OR “Charmosyna multistriata” OR “Charmosyna diadema” OR “Charmosyna amabilis” OR “Charmosyna margarethae” OR “Vini kuhlii” OR “Vini stepheni” OR “Vini peruviana” OR “Vini ultramarine” OR “Lorius garrulus” OR “Lorius domicella” OR “Lorius albidinucha” OR “Psitteuteles iris” OR “Eos histrio” OR “Eos reticulata” OR “Eos cyanogenia” OR “Trichoglossus forsteni” OR “Trichoglossus weberi” OR “Trichoglossus rosenbergii” OR “Trichoglossus johnstoniae” OR “Trichoglossus rubiginosus” OR “Lophornis gouldii” OR “Loriculus catamene” OR “Loriculus tener” OR “Loriculus exilis” OR “Loriculus pusillus” OR “Loriculus flosculus” OR “Elaenia ridleyana” OR “Notiomystis cincta” OR “Gymnomyza samoensis” OR “Gymnomyza aubryana” OR “Manorina melanotis” OR “Philemon brassi” OR “Philemon fuscicapillus” OR “Macgregoria pulchra” OR “Melidectes whitemanensis” OR “Melidectes princeps” OR “Lichmera notabilis” OR “Grantiella picta” OR “Myzomela kuehni” OR “Myzomela chermesina” OR “Myzomela malaitae” OR “Philesturnus carunculatus” OR “Vireo gracilirostris” OR “Zosterops mouroniensis” OR “Zosterops ficedulinus” OR “Zosterops griseovirescens” OR “Zosterops chloronothus” OR “Zosterops modestus” OR “Zosterops conspicillatus” OR “Zosterops rotensis” OR “Zosterops hypolais” OR “Zosterops natalis” OR “Zosterops flavus” OR “Zosterops grayi” OR “Zosterops uropygialis” OR “Zosterops somadikartai” OR “Zosterops nehrkorni” OR “Zosterops mysorensis” OR “Zosterops kuehni” OR “Zosterops vellalavella” OR “Zosterops splendidus” OR “Zosterops luteirostris” OR “Zosterops tenuirostris” OR “Zosterops albogularis” OR “Zosterops samoensis” OR “Zosterops oleaginous” OR “Woodfordia lacertosa” OR “Rukia longirostra” OR “Rukia ruki” OR “Cleptornis marchei” OR “Apalopteron familiar” OR “Madanga ruficollis” OR “Heleia muelleri” OR “Megazosterops palauensis” OR “Speirops melanocephalus” OR “Speirops brunneus” OR “Speirops leucophoeus” OR “Anthreptes reichenowi” OR “Anthreptes rhodolaemus” OR “Anthreptes pallidigaster” OR “Anthreptes rubritorques” OR “Nectarinia thomensis” OR “Nectarinia ursulae” OR “Nectarinia neergardi” OR “Nectarinia loveridgei” OR “Nectarinia moreaui” OR “Nectarinia rockefelleri” OR “Nectarinia rufipennis” OR “Aethopyga primigenia” OR “Aethopyga boltoni” OR “Aethopyga linaraborae” OR “Aethopyga duyvenbodei” OR “Hemignathus kauaiensis” OR “Hemignathus flavus” OR “Hemignathus parvus” OR “Hemignathus lucidus” OR “Hemignathus munroi” OR “Vestiaria coccinea” OR “Palmeria dolei” OR “Icterus oberi” OR “Icterus bonana” OR “Icterus laudabilis” OR “Thraupis cyanoptera” OR “Dacnis nigripes” OR “Conirostrum bicolor” OR “Diglossa venezuelensis” OR “Diglossa gloriosissima”)

AND TI= (“ecosystem servic*” OR “ecosystem function*” OR “pest regulat*” OR “disease regulat*” OR “pollin*” OR “nutrient cycl*” OR “soil form*” OR “climate regulat*” OR “natural-hazard regulat*” OR “water cycl*” OR “habitat provis*”)

AND TI= (“farm*” OR “agri*” OR “food”)

AND TI= ("contribut*" OR "produc*" OR "return*" OR "profit*" OR revenue OR return OR output OR "incom*" OR kg OR tonnes OR "dollar*" OR kcal OR calories OR hectare OR "ha" OR “ton*” OR “bushel*” OR crop OR "harvest*" OR yield)

NOT TI=(aquatic OR marine OR livestock OR cattle OR pig OR bushmeat OR “wetland*” OR “peatland*” OR “medicin*”)

## Search terms – Dutch

The Dutch search strings were constructed like the English search strings, the translated search terms are provided in Table S3.

Table S3: Search terms for systematic literature search on rare or endangered species’ contribution to agricultural production in Dutch. The asterisk (*) was used to find variations of words with a common root. For the advanced search strings, we connected entries of each column with ‘OR’ and combined columns with ‘AND’.

| **Rare/endangered** | | **Ecosystem service** | **Contribution to**  **agricultural production** |
| --- | --- | --- | --- |
| *IUCN*  zeldzaam  bedreigd*  kwetsbaar  gevoelig | | ecosysteemdienst*  ecosysteemfunctie*  natuurvoorde*  *regulating services*  klimaatregeling  regulering van natuurlijke gevaren  plaagbestrijding  natuurlijke bestrijding  ongediertebestrijding  ziektebestrijding  bestuiv*  *supporting services* nutriëntencycl* bodemvorm*  waterkringloop  natuurlijke leefomgeving | landbouw*  agrar*  levensmiddel*  voedingsmiddel*  voedsel  boederij *  *General contribution*  bijdrag*  produc*  inkom*  profijt*  opbrengst  winst  *Unit measure contribution*  kg  tonne*  $ / dollar  kcal / calorieën  ha / hektar  schepel  *Food contribution*  gewas  oogst*  opbrengst |
| gering*  klein*  weinig*  limit*  gelimit*  begrensd*  beperkt  afnemend*  dalend*  verlies | *Area*  gebied*  verspreidingsgebied*  verspreiding  distributie*  omvang*  niche  habitat    *Abundance*  abundantie  talrijkheid  frequentie  aantal  populatie*  individuen  dichtheid |  |  |
| *Species name-based search:*  Red list European bees (N=77)  Red list pollinating birds (N=169)  Red list pollinating mammals (N=101)  Red list pollinating insects of North America (N=111) | |  |  |

## Search terms – German

The German search strings were constructed like the English search strings, the translated search terms are provided in Table S4.

Table S4: Search terms for systematic literature search on rare or endangered species’ contribution to agricultural production in German. The asterisk (*) was used to find variations of words with a common root. For the advanced search strings, we connected entries of each column with ‘OR’ and combined columns with ‘AND’.

| **Rare/endangered** | | **Ecosystem service** | **Contribution to**  **agricultural production** |
| --- | --- | --- | --- |
| *IUCN*  selten*  gefährdet*  bedroht | | Ökosystemleistung* Ökosystemfunktion*  *regulating services*  Klimaregulation  Regulation von Naturgefahren  Schädlingsbekämpfung Krankheitsbekämpfung Bestäub*  *supporting services*  Nährstoffkreislauf Bodenbildung  Wasserzyklus  Lebensraum | Hof*  agrar*  Lebensmittel*  *General contribution*  beitag*  produzier*  Einnahme*  profit*  Revenue  output  Einkomm*  *Unit measure contribution*  kg  Tonne*  $ / Dollar  kcal / Kalorien  ha / Hektar  Buschel  *Food contribution*  Getreide  Feldfrucht  Ernte  Ertrag |
| gering*  klein*  wenig*  begrenzt*  abnehmend*  sinked*  fallend*  Verlust | *area*  Gebiet*  Verteilung  Ausbreitung  Ausdehnung  Nische  Habitat  *abundance*  Abundanz  Anzahl  Population*  Individuen  Dichte |  |  |
| *Species name-based search:*  Red list European bees (N=77)  Red list pollinating birds (N=169)  Red list pollinating mammals (N=101)  Red list pollinating insects of North America (N=111) | |  |  |

## Search terms – Spanish

The Spanish search strings were constructed like the English search strings, the translated search terms are provided in Table S5.

Table S5: Search terms for systematic literature search on rare or endangered species’ contribution to agricultural production in Spanish. The asterisk (*) was used to find variations of words with a common root. For the advanced search strings, we connected entries of each column with ‘OR’ and combined columns with ‘AND’.

| **Rare/endangered** | | **Ecosystem service** | **Contribution to**  **agricultural production** |
| --- | --- | --- | --- |
| *IUCN*  rara*  peligro de extinction*  vulnerable*  amenazada*  en peligr* | | servicios ecosistémicos servicios ambientales funcionamiento del ecosistema    *regulating services*  regulación del clima  regulación de riesgos naturales  control de plagas  control de enfermedades  polinización*  *supporting services*  ciclo de nutrients  formación del suelo  ciclo del agua  provisión de hábitat | granja*  alquería*  agrario*  alimentos*  comida    *General contribution*  contribución*  producción*  retorno*  beneficio*  rédito*  retorno*  ingresos    *Unit measure contribution*  kg  toneladas*  $ / dollar  kcal / caloric  ha / hectárea  fanega*    *Food contribution*  cultivo  cosecha  vendimia |
| escasa/ poco común*  pequeña*  limitada*  restricta*  en declive*  en regresión*  pérdida* | *Area*  área*  distribución*  extensión*  nicho  habitat    *abundance*  abundant*  cantidad*  población*  individuos  densidad |  |  |
| *Species name-based search:*  Red list European bees (N=77)  Red list pollinating birds (N=169)  Red list pollinating mammals (N=101)  Red list pollinating insects of North America (N=111) | |  |  |

## Search terms – French

The French search strings were constructed like the English search strings, the translated search terms are provided in Table S6.

Table S6: Search terms for systematic literature search on rare or endangered species’ contribution to agricultural production in French. The asterisk (*) was used to find variations of words with a common root. For the advanced search strings, we connected entries of each column with ‘OR’ and combined columns with ‘AND’.

| **Rare/endangered** | | **Ecosystem service** | **Contribution to**  **agricultural production** |
| --- | --- | --- | --- |
| *IUCN*  rare  menacé*  vulnérable  en danger | | service écosystémique  fonctionnement des écosystèmes  *supporting services*  régulation du climat  Régulation des effets des risques naturels  régulation des populations de ravageurs  régulation des maladies  pollinis*  *regulating services*  cycle des nutriments  formation de* sol*  cycle de l'eau  offre d'habitat | ferm*  agri*  agro*  nourriture  aliment*  *General contribution*  contribut*  produc*  retour*  profit*  revenu  *Unit measure contribution*  kg  ton*  $ / dollar  kcal / calories  ha / hectare  boisseau  *Food contribution*  culture  récolte*  rendement* |
| faible  petit*  peu de  limit*  restr*  confin*  declin*  diminu*  tombant*  chut*  perte  dispar* | *area*  distribution  mesure  niche  habitat  espace  *abundance*  abondance  nombre*  population*  individu*  densité |  |  |
| *Species name-based search:*  Red list European bees (N=77)  Red list pollinating birds (N=169)  Red list pollinating mammals (N=101)  Red list pollinating insects of North America (N=111) | |  |  |

# **S4 Reference screening**

Table S7: Articles that we considered for the extended search and number of additional relevant articles using Google Scholar profiles.

| **Relevant article** | **Additional relevant articles** |
| --- | --- |
| Gera Hol et al. 2015 | 0 |
| Kleijn et al. 2015 | 0 |
| Klein et al. 2003 | 1 (Kremen et al. 2002) |
| Kremen et al. 2002 | 0 |
| Kross et al. 2012 | 0 |
| Larsen et al. 2005 | 0 |
| MacLeod et al. 2020 | 1 (Soliveres et al. 2016) |
| Soliveres et al. 2016 | 0 |
| Sutter et al. 2017 | 0 |
| Twerski et al. 2021 | 0 |
| Winfree et al. 2015 | 0 |
| Winfree et al. 2018 | 0 |

# **References**

Chen, Q.-L., Ding, J., Zhu, D., Hu, H.-W., Delgado-Baquerizo, M., Ma, Y.-B., He, J.-Z., & Zhu, Y.-G. (2020). Rare microbial taxa as the major drivers of ecosystem multifunctionality in long-term fertilized soils. *Soil Biology and Biochemistry*, *141*, 107686. https://doi.org/10.1016/j.soilbio.2019.107686

Deeks, J. J., Higgins, J. P., Altman, D. G., & Group, on behalf of the C. S. M. (2019). Analysing data and undertaking meta-analyses. In *Cochrane Handbook for Systematic Reviews of Interventions* (pp. 241–284). John Wiley & Sons, Ltd. https://doi.org/10.1002/9781119536604.ch10

DuVal, A., Mijatovic, D., & Hodgkin, T. (2019). *The contribution of biodiversity for food and agriculture to the resilience of production systems –Thematic Study for The State of the World’s Biodiversity for Food and Agriculture.* FAO.

Fox, J. W. (2006). Using the Price Equation to partition the effects of biodiversity loss on ecosystem function. *Ecology*, *87*(11), 2687–2696. https://doi.org/10.1890/0012-9658(2006)87[2687:UTPETP]2.0.CO;2

Hedrzak, M. J., Badach, E., & Kornaś, S. A. (2021). Preliminary assumptions for identification of the common hamster (Cricetus cricetus) as a service provider in the agricultural ecosystem. *Sustainability (Switzerland)*, *13*(12). https://doi.org/10.3390/su13126793

IUCN. (2021). *The IUCN Red List of Threatened Species. Version 2021-1*. https://www.iucnredlist.org/

King, C., Ballantyne, G., & Willmer, P. G. (2013). Why flower visitation is a poor proxy for pollination: Measuring single-visit pollen deposition, with implications for pollination networks and conservation. *Methods in Ecology and Evolution*, *4*(9), 811–818. https://doi.org/10.1111/2041-210X.12074

Kleijn, D., Winfree, R., Bartomeus, I., Carvalheiro, L. G., Henry, M., Isaacs, R., Klein, A.-M., Kremen, C., M’Gonigle, L. K., Rader, R., Ricketts, T. H., Williams, N. M., Lee Adamson, N., Ascher, J. S., Báldi, A., Batáry, P., Benjamin, F., Biesmeijer, J. C., Blitzer, E. J., … Potts, S. G. (2015). Delivery of crop pollination services is an insufficient argument for wild pollinator conservation. *Nature Communications*, *6*, 7414.

Klein, A.-M., Steffan-Dewenter, I., & Tscharntke, T. (2003). Fruit set of highland coffee increases with the diversity of pollinating bees. *Proceedings of the Royal Society B: Biological Sciences*, *270*(1518), 955–961. https://doi.org/10.1098/rspb.2002.2306

Kremen, C., Williams, N. M., & Thorp, R. W. (2002). Crop pollination from native bees at risk from agricultural intensification. *Proceedings of the National Academy of Sciences*, *99*(26), 16812. https://doi.org/10.1073/pnas.262413599

Kross, S. M., Tylianakis, J. M., & Nelson, X. J. (2012). Effects of Introducing Threatened Falcons into Vineyards on Abundance of Passeriformes and Bird Damage to Grapes. *Conservation Biology*, *26*(1), 142–149. https://doi.org/10.1111/j.1523-1739.2011.01756.x

Larsen, T. H., Williams, N. M., & Kremen, C. (2005). Extinction order and altered community structure rapidly disrupt ecosystem functioning. *Ecology Letters*, *8*(5), 538–547. https://doi.org/10.1111/j.1461-0248.2005.00749.x

MacLeod, M., Reilly, J., Cariveau, D. P., Genung, M. A., Roswell, M., Gibbs, J., & Winfree, R. (2020). How much do rare and crop-pollinating bees overlap in identity and flower preferences? *Journal of Applied Ecology*, *57*(2), 413–423. https://doi.org/10.1111/1365-2664.13543

National Research Council. (2007). *Status of Pollinators in North America*. The National Academies Press. https://doi.org/10.17226/11761

Nieto, A., Roberts, S. P. M., Kemp, J., Rasmont, P., Kuhlmann, M., García Criado, M., Biesmeijer, J. C., Bogusch, P., Dathe, H. H., De la Rúa, P., De Meulemeester, T., Dehon, M., Dewulf, A., Ortiz-Sánchez, F. J., Lhomme, P., Pauly, A., Potts, S. G., Praz, C., Quaranta, M., … Michez, D. (2014). *European Red List of bees*.

Pullin, A. S., & Stewart, G. B. (2006). Guidelines for Systematic Review in Conservation and Environmental Management. *Conservation Biology*, *20*(6), 1647–1656. https://doi.org/10.1111/j.1523-1739.2006.00485.x

Regan, E. C., Santini, L., Ingwall-King, L., Hoffmann, M., Rondinini, C., Symes, A., Taylor, J., & Butchart, S. H. M. (2015). Global Trends in the Status of Bird and Mammal Pollinators. *Conservation Letters*, *8*(6), 397–403. https://doi.org/10.1111/conl.12162

Reid, W., Mooney, H., Cropper, A., Capistrano, D., Carpenter, S., Chopra, K., Dasgupta, P., Dietz, T., Duraiappah, A., Hassan, R., Kasperson, R., Leemans, R., May, R., Mcmichael, A., Pingali, P., Samper, C., Scholes, R., Watson, R., Zakri, A. H., & Zurek, M. (2005). *Millenium Ecosystem Assessment Synthesis Report*.

Simpson, D., Weinman, L., Genung, M., Roswell, M., Macleod, M., & Winfree, R. (2022). Many bee species, including rare species, are important for function of entire plant-pollinator networks. *Proceedings of the Royal Society B: Biological Sciences*, *289*. https://doi.org/10.1098/rspb.2021.2689

Soliveres, S., Manning, P., Prati, D., Gossner, M. M., Alt, F., Arndt, H., Baumgartner, V., Binkenstein, J., Birkhofer, K., Blaser, S., Blüthgen, N., Boch, S., Böhm, S., Börschig, C., Buscot, F., Diekötter, T., Heinze, J., Hölzel, N., Jung, K., … Allan, E. (2016). Locally rare species influence grassland ecosystem multifunctionality. *Philosophical Transactions of the Royal Society B: Biological Sciences*, *371*(1694), 20150269. https://doi.org/10.1098/rstb.2015.0269

Staton, T., Walters, R. J., Breeze, T. D., Smith, J., & Girling, R. D. (2022). Niche complementarity drives increases in pollinator functional diversity in diversified agroforestry systems. *AGRICULTURE ECOSYSTEMS & ENVIRONMENT*, *336*. https://doi.org/10.1016/j.agee.2022.108035

Sutter, L., Jeanneret, P., Bartual, A. M., Bocci, G., & Albrecht, M. (2017). Enhancing plant diversity in agricultural landscapes promotes both rare bees and dominant crop-pollinating bees through complementary increase in key floral resources. *Journal of Applied Ecology*, *54*(6), 1856–1864. https://doi.org/10.1111/1365-2664.12907

Winfree, R., Fox, J. W., Williams, N. M., Reilly, J. R., & Cariveau, D. P. (2015). Abundance of common species, not species richness, drives delivery of a real-world ecosystem service. *Ecology Letters*, *18*(7), 626–635. https://doi.org/10.1111/ele.12424

Winfree, R., Reilly, J. R., Bartomeus, I., Cariveau, D. P., Williams, N. M., & Gibbs, J. (2018). Species turnover promotes the importance of bee diversity for crop pollination at regional scales. *Science*, *359*(6377), 791–793. https://doi.org/10.1126/science.aao2117

Yanai, R. D., Mann, T. A., Hong, S. D., Pu, G., & Zukswert, J. M. (2021). The current state of uncertainty reporting in ecosystem studies: A systematic evaluation of peer-reviewed literature. *Ecosphere*, *12*(6), e03535. https://doi.org/10.1002/ecs2.3535

Zhang, Z., Lu, Y., Wei, G., & Jiao, S. (2022). Rare Species-Driven Diversity-Ecosystem Multifunctionality Relationships are Promoted by Stochastic Community Assembly. *MBIO*, *13*(3). https://doi.org/10.1128/mbio.00449-22
